# Postoperative Recurrence in Crohn’s Disease: Pathophysiology, Risk Stratification, and Management Strategies

**DOI:** 10.3390/jcm15010243

**Published:** 2025-12-28

**Authors:** Luisa Bertin, Gianluca Semprucci, Camilla Cavagna, Miriana Zanconato, Marco Scarpa, Cesare Ruffolo, Imerio Angriman, Andrea Buda, Gaia Riguccio, Fabiana Zingone, Brigida Barberio, Edoardo Vincenzo Savarino

**Affiliations:** 1 Gastroenterology Unit, Department of Surgery, Oncology and Gastroenterology, University of Padova, 35128 Padova, Italy; luisa.bertin.1@phd.unipd.it (L.B.); miriana.zanconato@studenti.unipd.it (M.Z.);; 2 Chirurgia Generale 3 Unit, Azienda Ospedale Università di Padova, Via Giustiniani 2, 35128 Padua, Italycesare.ruffolo@aopd.veneto.it (C.R.);; 3 Gastroenterology Unit, Department of Oncological Gastrointestinal Surgery, S. Maria del Prato Hospital, 32032 Feltre, Italy; andrea.buda@aulss1.veneto.it (A.B.);

**Keywords:** Crohn’s disease, postoperative recurrence, ileocolic resection, endoscopic recurrence, biologic therapy, anti-TNF agents, risk stratification, Rutgeerts score, fecal calprotectin, intestinal ultrasound

## Abstract

Postoperative recurrence (POR) remains a significant challenge in Crohn’s disease (CD) management despite therapeutic advances. Contemporary data show ileocecal resection rates of 18.7%, 28.0%, and 39.5% at one, five, and ten years after diagnosis, with endoscopic recurrence occurring in 22.4–53% of patients within 18–36 months postoperatively. Current understanding of POR pathophysiology includes microbiota dysbiosis, mesenteric inflammation, immune dysregulation, and genetic factors, particularly NOD2 variants. Key risk factors comprehend smoking, penetrating or perianal disease, prior surgeries, and extensive small bowel involvement. The Rutgeerts score remains the endoscopic gold standard for assessing recurrence, though it has never been validated and modifications addressing modern anastomotic techniques have been proposed. Non-invasive monitoring strategies using fecal calprotectin, intestinal ultrasound, and magnetic resonance enterography demonstrate promising diagnostic performance and may reduce the burden of routine endoscopy. Anti-TNF agents and Vedolizumab show superior efficacy in preventing endoscopic recurrence compared to conventional therapies, while other advanced therapies like anti-JAKs, risankizumab and ustekinumab demonstrate potential benefit in postoperative prophylaxis. Management approaches have evolved toward risk-stratified strategies balancing systematic prophylaxis against endoscopy-driven therapy. While medical prophylaxis remains first-line for high-risk patients, the expanding therapeutic armamentarium and improved understanding of pathophysiologic mechanisms enable increasingly personalized postoperative care. Further research is needed to validate risk assessment tools, optimize timing and selection of prophylactic therapies, and define the role of emerging agents in reducing long-term disease burden.

## 1. Introduction

Crohn’s Disease (CD) is a chronic, disabling inflammatory disorder that significantly impacts both individual health and the healthcare system. Before the widespread use of biologic therapies, a meta-analysis reported that the likelihood of surgical ileocecal resection following a CD diagnosis was 16.3% at one year, 33.3% at five years, and 46.6% at ten years [1,2]. With improvements in early diagnosis, the implementation of advanced immunological treatments and tight disease monitoring have reduced the long-term need for surgery. Indeed, a recent meta-analysis of patients diagnosed with Crohn’s disease after 2000 showed ileocecal resection rates of 18.7%, 28.0%, and 39.5% at one, five, and ten years, respectively [3]. However, surgical intervention remains an essential treatment option in many cases.

The Postoperative Crohn’s Endoscopic Recurrence (POCER) trial established the critical value of early endoscopic assessments and risk stratification in guiding therapeutic decisions, forming the basis for current clinical guidelines [4,5,6,7,8]. While infliximab and Adalimumab have demonstrated substantial efficacy in mitigating endoscopic postoperative recurrence (POR), the growing availability of new biologic treatments has spurred research into newer agents such as ustekinumab and vedolizumab. A dynamic, evidence-based approach to the identification and prevention of POR is imperative, as its progression spans a continuum from microscopic inflammatory activity to severe complications necessitating further surgery [9]. Despite these advances, several critical challenges persist in optimizing postoperative outcomes. The precise mechanisms driving POR remain incompletely understood, though research has identified several contributing domains including intestinal microbiota dysbiosis, mesenteric inflammation, immune dysregulation, and genetic susceptibility. Understanding these pathophysiologic processes is essential for developing targeted preventive strategies and identifying patients at highest recurrence risk.

Furthermore, while infliximab, adalimumab and vedolizumab have demonstrated substantial efficacy in reducing endoscopic recurrence, the expanding therapeutic armamentarium including newer biologic agents such as ustekinumab, risankizumab and anti-JAKs require systematic evaluation in the postoperative setting. The optimal approach to risk stratification, the comparative effectiveness of various prophylactic regimens, and strategies for managing patients with prior biologic exposure all remain areas requiring further evidence synthesis and clinical guidance.

## 2. Underlying Mechanisms and Contributing Factors

**Figure 1:**
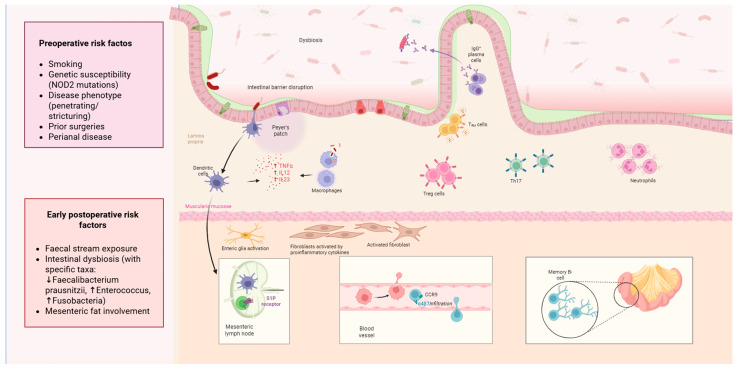
Pathophysiological mechanisms of post-operative recurrence after surgical resection in patients with Crohn’s disease.

Subsequent to the seminal work of Rutgeerts et al., the pathophysiology of POR in CD has been the subject of extensive investigation [10]. However, the precise mechanisms driving this phenomenon remain undetermined. Research efforts have primarily concentrated on four key areas of inquiry: the microbiome, the mesentery, the immune system and genetics. However, the exact mechanisms have remained elusive. An overview of the pathophysiological mechanisms of post-operative recurrence is illustrated in Figure 1.

### 2.1. Microbiota

Landmark studies conducted over two decades ago identified the intestinal contents’ role in triggering POR. These investigations showed that when the fecal stream was diverted, no inflammation occurred in the neoterminal ileum. However, exposure to luminal content resulted in rapid microscopic inflammation, demonstrating the intestinal contents causal role in POR [11]. The specific factors responsible remain undefined, but potential culprits include microbial dysbiosis, bile acids, or dietary influences. Additional research has shown that antibiotics like nitroimidazoles can reduce POR risk, with a relative risk (RR) of 0.23 for clinical recurrence and 0.44 for endoscopic recurrence, though their long-term use is limited by adverse events [12]. The gut microbiome—encompassing not only the bacteriome but also the virome and mycobiome—plays a central role in maintaining intestinal homeostasis, and its disruption has been increasingly recognized as a key contributor to IBD pathogenesis and recurrence. In healthy individuals, commensal bacteria produce short-chain fatty acids (SCFAs) such as butyrate, which exert anti-inflammatory effects by inhibiting histone deacetylase activity and suppressing NF-κB activation, thereby promoting regulatory T cell differentiation and maintaining epithelial barrier integrity. Dysbiosis in CD is characterized by reduced abundance of butyrate-producing obligate anaerobes (particularly *Faecalibacterium prausnitzii* and *Ruminococcaceae*) and expansion of facultative anaerobes such as Proteobacteria. This microbial imbalance leads to decreased SCFA production, thinning of the mucus layer, increased intestinal permeability, and activation of Th1/Th17 inflammatory pathways [13]. Importantly, dysbiosis extends beyond the bacteriome: alterations in the gut virome (particularly Caudovirales bacteriophages) and mycobiome (with increased Candida species and decreased Saccharomyces cerevisiae) have also been implicated in IBD progression [new reference]. In the postoperative setting, surgical manipulation and fecal stream diversion may further exacerbate these dysbiotic changes, creating a pro-inflammatory milieu at the anastomotic site that favors disease recurrence. Specific microbial shifts, such as reduced *Faecalibacterium prausnitzii* linked to decreased IL-10 secretion and increased IL-12 and interferon-gamma (IFN γ) levels, have been associated with higher recurrence rates [14]. Emerging studies have also noted a significant presence of *Enterococcus durans* and *Fusobacteria* in cases of POR, beside exploratory clinical trials aimed at microbiota modulation [15,16,17,18,19].

### 2.2. The Role of Mesentery

Creeping fat, a hallmark feature of CD, involves hypertrophic mesenteric adipose tissue encircling inflamed bowel segments. Evidence suggests that the mesentery is a central player in initiating and sustaining inflammation, potentially acting as a primary trigger for CD [20,21,22]. The degree of mesenteric involvement correlates with the severity of local inflammation, as observed in imaging and histological analyses [23]. The role of the mesentery remains ambiguous, potentially being both protective and detrimental [24,25,26,27,28].

Mesenteric lymph nodes may represent the anatomical reservoir of immune memory in CD. These nodes are enriched in Th17 memory T-cells, particularly effector memory cells, which can be activated through IL-12-dependent pathways and switched to a Th1 phenotype before homing back to the mucosa [29]. Enhancement of mesenteric lymph nodes on imaging studies predicts active disease, supporting their central role in memory-driven immune reactivation [30].

Surgical strategies incorporating mesenteric resection during ileocecal resection, while employing a less radical approach compared to those utilized in colorectal cancer surgery, have been postulated to diminish the incidence of POR [31,32]. However, a recent RCT showed that extended mesenteric resection was not superior to conventional resection with regard to endoscopic POR [33,34]. Thus, more studies are needed to clarify the role of mesentery in the pathophysiology of disease recurrence.

### 2.3. Immune Dynamics

The immune system plays a pivotal role in POR pathogenesis, with memory T-lymphocytes retained in mesenteric lymph nodes representing a key driving force [35,36]. Recent evidence from a prospective cohort study of 88 patients undergoing ileocolonic resection provides additional insights into the temporal evolution of innate immunity in CD [37]. In newly diagnosed patients, elevated levels of IL-15 and IL-23 in histologically healthy ileal mucosa suggest that innate immune activation serves as an initiator of acute inflammation. Notably, M2 macrophages progressively increase in the healthy mucosa of patients with late-stage disease, indicating a shift toward reparative and profibrotic processes over time. This temporal transition may explain why anti-inflammatory therapies demonstrate reduced efficacy in long-standing disease, supporting the rationale for early surgical intervention in appropriately selected patients. Cell-mediated immunity appears central to the recurrence process. Significant findings by the REMIND study group underscore the involvement of cytotoxic CD8+ T cells, also known as cytotoxic T lymphocytes (CTLs), enriched in granzyme B and perforins, in driving POR. Higher clonal frequencies of T-cell receptors (TCRs) have been observed in patients with recurrence, with smoking identified as a significant contributor to this immune dysregulation [38]. Notably, postoperative persistence of a T-cell receptor (TCR) repertoire similar to pre-resection patterns correlates with endoscopic recurrence development.

The CD4+NKG2D+ T-cell subset at ileal mucosal margins has been identified as predictive of endoscopic recurrence, displaying high expression of TNF-α and other pro-inflammatory molecules [39]. The persistence of subclinical intestinal inflammation following surgery plays a crucial role in driving systemic inflammatory responses and subsequent recurrence. Even in patients achieving clinical remission after bowel resection, elevated fecal lactoferrin levels—a direct marker of intestinal inflammation—correlate significantly with serum IL-6 (R = 0.431, *p* = 0.025) and CRP (R = 0.507, *p* = 0.007), suggesting an ongoing IL-6-CRP inflammatory cascade [40]. Notably, higher IL-6 levels predict increased risk of reoperation for anastomotic recurrence (*p* = 0.10), indicating that subclinical intestinal inflammation maintains a ‘smoldering fire’ of systemic inflammation that predisposes to clinical recurrence. Cytokine dysregulation contributes substantially to recurrence. Elevated mucosal IL-6 levels correlate with clinical recurrence, while reduced mucosal IL-10 predicts endoscopic recurrence development [41]. Elevated levels of inflammatory cytokines such as IL-1β, TNF-α, and IL-6 have been detected in the mucosa of recurrent cases [42]. Enteric glial cells expressing S100 proteins have also been implicated as risk markers for both clinical and endoscopic recurrence [43].

Beyond its role in active inflammation, aberrant wound healing and fibrogenic processes in macroscopically healthy bowel may predispose to recurrence. TGF-β1, a key regulator of fibrogenesis and tissue repair, demonstrates prognostic significance when measured in histologically normal ileal segments at the time of resection. Patients with elevated [44] TGF-β1 mRNA expression in healthy bowel exhibit significantly higher cumulative recurrence rates compared to those with low expression levels (*p* = 0.02), with TGF-β1 levels showing direct correlation with clinical recurrence (τ = 0.43, *p* = 0.04). Notably, IGF-1 expression shows no association with recurrence risk, suggesting specificity of the TGF-β1 pathway in POR pathogenesis.

MicroRNAs (miRNAs) have emerged as important epigenetic regulators linking genetic susceptibility, immune activation, and tissue remodeling in CD. These small non-coding RNAs modulate gene expression at the post-transcriptional level and participate in processes central to POR pathogenesis, including inflammation, apoptosis, autophagy, epithelial barrier function, and fibrogenesis.

Recent studies have identified specific miRNA signatures with potential predictive value for postoperative recurrence. Moret-Tatay et al. demonstrated that a plasma miRNA signature (miR-191-5p, miR-15b-5p, miR-106b-5p, miR-451a, and miR-93-5p) could predict POR at the time of surgery with an AUC of 0.88, while another signature (miR-15b-5p, miR-451a, miR-93-5p, miR-423-5p, and miR-125b-5p) confirmed recurrence within one year with an AUC of 0.96 [45]. These miRNAs regulate pathways involved in TNF signaling, apoptosis resistance, reactive oxygen species metabolism, and pro-inflammatory T-cell differentiation.

At the tissue level, Steigleder et al. validated the overexpression of miRNA-650 and miRNA-29c in the mesenteric adipose tissue of CD patients undergoing surgery, with corresponding downregulation of their target genes involved in amino acid metabolism (GFPT2, ALDH4A1), cell cycle regulation (E2F1), hypoxia response (HIF3A), energy metabolism (PDK4), and lipid storage (CIDEC) [46]. Notably, these investigators developed a mathematical model incorporating miRNA levels and clinical variables that predicted the time to postoperative relapse with high accuracy, representing a potential tool for guiding individualized postoperative management.

These findings suggest that miRNA profiling—whether in plasma or mesenteric adipose tissue—may serve as a biomarker-based approach to risk stratification, complementing clinical factors in identifying patients at highest risk of early recurrence who may benefit from intensified prophylactic therapy.

### 2.4. Genetics

A recent systematic review and meta-analysis examined the role of genetic factors in POR of CD [47]. Among 28 studies including 6715 patients, 13 loci were identified as influencing recurrence risk, with the NOD2 gene being the most strongly associated. Patients carrying the NOD2 risk allele had 1.64-fold higher odds of recurrence compared to those without (*p* = 0.003). While other genes such as BACH2, CARD8, SMAD3 and TNFSF15 were implicated in single studies, their roles remain less substantiated. The RNASET2 gene, involved in IFNγ production enhancement, associates with more severe recurrence (Rutgeerts score > 2) and shorter time to repeat surgery [48]. The findings emphasize the need for standardized reporting in future genetic studies to better elucidate POR mechanisms and inform tailored therapies.

### 2.5. Emerging Mechanistic Pathways

Beyond the established mechanisms discussed above, emerging evidence implicates additional pathways in POR pathogenesis. The gut–brain axis, through stress-induced activation of the hypothalamic–pituitary–adrenal axis, may influence intestinal permeability, mucosal immunity, and microbiome composition, potentially affecting postoperative outcomes [49]. Whether perioperative stress-reducing interventions could complement medical prophylaxis warrants investigation.

Intestinal barrier dysfunction, characterized by altered tight junction proteins and reduced mucus layer integrity, permits bacterial translocation and perpetuates inflammation. Surgical resection removes diseased tissue but does not correct underlying barrier abnormalities. Emerging therapeutic strategies targeting barrier restoration, including specific probiotics and butyrate supplementation, may eventually complement immunosuppressive approaches [50,51,52]. Future predictive models may benefit from integrating biomarkers reflecting these pathways alongside traditional clinical risk factors.

## 3. Diagnosis and Natural History of Postoperative Recurrence

### 3.1. Histologic Postoperative Recurrence

**Figure 2:**
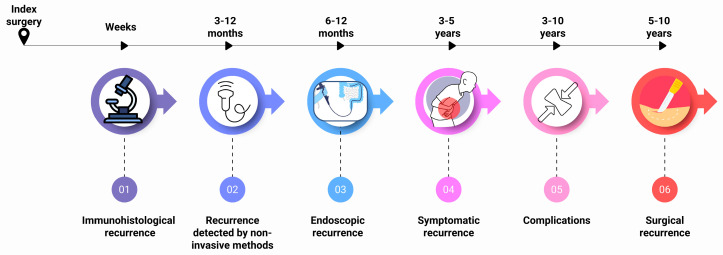
Natural history of post-operative recurrence after surgical resection in patients with Crohn’s disease.

POR of CD frequently manifests in the neoterminal ileum and ileocolonic anastomosis, representing a continuum from histological activity to clinical complications necessitating further surgical intervention. Histologic recurrence is often detected as early as one week after ileocecal resection [11]. This recurrence can occur even in the absence of endoscopic findings and has been identified as a predictor of subsequent endoscopic disease progression, thus highlighting its potential role in stratifying patients at higher risk for clinical worsening [53]. Ileocecal resection remains the most studied surgical intervention in CD, further emphasizing the importance of monitoring early histologic changes to optimize postoperative management. A timeline of the natural history of POR is illustrated in Figure 2.

### 3.2. Recurrence Detected by Non-Invasive Methods

Several non-invasive diagnostic tools have emerged as valuable alternatives or complements to standard ileocolonoscopy for detecting postoperative recurrence. These methods, including fecal calprotectin, intestinal ultrasound, and cross-sectional imaging, can identify mucosal and transmural changes without the burden of endoscopic procedures. Fecal calprotectin (FC) demonstrates pooled sensitivity of 82% and specificity of 61% for endoscopic recurrence, with cutoffs < 50 μg/g.

Potentially obviating routine endoscopy in low-risk patients [54]. The advent of cross-sectional imaging techniques has provided a non-invasive means of assessing disease activity in both preoperative and postoperative contexts for CD. Cross-sectional imaging provides unique value by detecting transmural disease activity even when endoscopic findings are unremarkable, identifying patients at higher risk for disease progression [55].

Beyond traditional inflammatory markers, lipid metabolism alterations may reflect disease activity in the postoperative period [56]. Following intestinal resection, patients demonstrate improved inflammatory status accompanied by favorable changes in lipid metabolism, including increased HDL cholesterol levels (*p* = 0.02). Notably, disease recurrence reverses these improvements, with patients experiencing active recurrent disease showing significantly lower total cholesterol (*p* < 0.01), HDL (*p* = 0.01), and LDL cholesterol (*p* = 0.01) compared to those maintaining remission. These lipid alterations correlate with disease activity rather than the extent of bowel resection, suggesting that systemic metabolic derangements may serve as indirect indicators of inflammatory recurrence.

Computed tomography enterography (CTE) and MRE have shown excellent accuracy in detecting inflammation associated with CD, achieving high levels of sensitivity and specificity when compared to endoscopic findings [57]. A systematic review and meta-analysis by Chavoshi and colleagues (2024) including 589 patients demonstrated that MR enterography and CT enterography achieved pooled sensitivities of 90% and 93%, respectively, with MRE showing superior specificity (78% versus 67%) for detecting post-operative CD recurrence after ileocecal resection. A significant advancement in radiologic evaluation is the MRE-based MONITOR index, which provides a validated tool for identifying endoscopic POR. Using a threshold of MONITOR index ≥ 1, this method demonstrated sensitivity of 87%, specificity of 75%, positive predictive value (PPV) of 84.6%, and negative predictive value (NPV) of 75% for identifying endoscopic POR classified as Rutgeerts score ≥ i2 [58].

Key imaging features associated with recurrence include wall thickening, anastomosis stenosis, penetrating lesions, and comb sign. These radiographic signs may signal a greater risk of disease progression than observed in patients who achieve remission in both modalities. These may signal a greater risk of disease progression than observed in patients who achieve remission in both modalities [59,60]. Additionally, intestinal ultrasound (IUS) has gained recognition as a reliable imaging modality for detecting endoscopic POR, offering strong sensitivity and specificity for disease monitoring [59,60,61]. Intestinal ultrasound shows excellent diagnostic performance with sensitivity of 94% and specificity of 84% for detecting recurrence, while magnetic resonance enterography (MRE) achieves 97% sensitivity and 84% specificity [62]. This evolution in imaging technology provides clinicians with complementary tools for early identification and risk stratification of POR in CD.

### 3.3. Endoscopic Postoperative Recurrence and the Role of Scoring Systems

Endoscopy remains the definitive tool for identifying POR in CD. Endoscopic recurrence is observed in a substantial proportion of patients within the first year post-surgery, ranging from 70–90% in pre-biologic eras to lower rates of 22.4–53% in the biologic era over 18–36 months [9,63,64,65,66,67,68].

The Rutgeerts score is the traditional tool for assessing mucosal healing or inflammation in the neoterminal ileum and categorizes findings from normal (i0) to severe inflammation with complications (i4) [10]. This scoring system has demonstrated prognostic value; for instance, patients with scores of i0–i1 are less likely to experience progression over three years, whereas those with scores of i3–i4 face a high probability of severe disease progression [10]. Although the clinical significance of i2 lesions remains debated, findings consistently indicate that ileal lesions carry a higher risk for adverse outcomes and require intensified monitoring and treatment [69,70,71,72]. Comparative studies between Rutgeerts, Simple Endoscopic Score for Crohn’s Disease (SES-CD), and modified SES-CD scores indicate similar predictive accuracy, with the latter offering additional utility in capturing colonic disease recurrence [73].

A notable limitation of the Rutgeerts score is its moderate inter-observer reliability. Multiple independent studies have demonstrated low agreement between endoscopists, including in the clinically important distinction between lesions < i2 and ≥i2, potentially leading to suboptimal therapeutic decisions in up to 10% of patients. The IOIBD consensus conference recommended that inter-observer agreement could be improved through training programs, and that centralized reading with good-quality videos should be considered for clinical trials [74].

Modifications to the Rutgeerts score, differentiating i2a (lesions confined to the anastomosis) from i2b (lesions involving the neoterminal ileum), aim to refine risk stratification and guide therapeutic interventions [55]. Recent data suggest that distinction between i2a and i2b lesions provides better insight into the progression of disease, with i2b lesions being more likely to advance to severe endoscopic POR (≥i3) [69,70,71,72].

The traditional Rutgeerts score has limitations, particularly in its application to modern surgical configurations, as it was proposed when the primary anastomosis technique was hand-sewn end-to-end anastomoses. Moreover, inflammation localized to ileal blind loops, not accounted for in the original Rutgeerts score, has been associated with increased risk of progression [75,76]. The proposed updated Rutgeerts score expand lesion categorization to incorporate distinct anastomotic configurations introduced by newer surgical techniques like side-to-side and side-to-end anastomoses [77] and the evaluation of the ileal blind loop. These refinements reflect the evolution of surgical practices and emphasize the need for dynamic endoscopic assessment frameworks. Additional scoring methods, such as the REMIND score, separately evaluate anastomotic and ileal lesions to better predict clinical outcomes [78]. The Rutgeerts score and its subsequent modifications, as well as the REMIND score, are presented in Table 1.

### 3.4. Clinical Postoperative Recurrence

Historical data suggests that approximately half of patients undergoing ileocecal resection experience clinical recurrence within five years of surgery [79]. While clinical symptoms often appear later than endoscopic findings, understanding the underlying cause of symptoms in the postoperative period is vital.

Importantly, clinical symptoms in the postoperative setting may not always reflect CD recurrence but rather anatomical and functional sequelae of intestinal resection. The CDAI, while useful in active luminal CD, shows poor correlation with POR. Symptoms such as diarrhea may result from bile acid malabsorption (BAM), reduced absorptive capacity, or altered motility rather than inflammatory recurrence. Moreover, surgical removal of the ileocecal valve frequently promotes bacterial overgrowth in the intestine. These conditions are recognized as potential causes of persistent diarrhea and should not automatically be attributed to POR. They are particularly likely when diarrhea develops soon after surgery (typically within a few days) without accompanying symptoms such as abdominal pain, fever, or elevated inflammatory biomarkers. In such situations, empirical administration of cholestyramine may be beneficial.

Moreover, patients with CD may exhibit symptoms that resemble irritable bowel syndrome (IBS), complicating the interpretation of digestive complaints [80,81,82]. Therefore, the identification of clinical POR requires more than symptom evaluation and should include morphological imaging to ensure an accurate diagnosis.

Relying solely on clinical symptoms to define recurrence and adapt treatment exposes patients to risk of under- or over-treatment. POR suspected by symptoms should be confirmed by endoscopy and/or cross-sectional imaging [74]. Moreover, patient-reported outcomes and quality of life measures should be part of postoperative follow-up. Long-term functional consequences of surgery, including impaired peristalsis, malabsorption, BAM, and vitamin B12 deficiency, may significantly impact quality of life independent of inflammatory recurrence. These symptoms may be better correlated with extent and site of resection than with endoscopic recurrence itself.

Estimates indicate that 30–60% of individuals develop symptomatic POR within 3–5 years of surgery [83,84,85]. In contrast to endoscopic POR, the impact of biologic therapies on reducing clinical recurrence remains inconclusive [9,63,86,87]. Notably, radiologic, and endoscopic evidence of recurrence do not always align with the presence of clinical symptoms [88,89]. For example, Rutgeerts et al. found that while 73% of patients exhibited endoscopic POR within the first postoperative year, only about 20% reported clinical symptoms during the same period [10,83]. However, the severity of endoscopic POR, particularly cases classified as ≥i3, correlates strongly with clinical manifestations, emphasizing the importance of treating endoscopic recurrence to prevent progression and the onset of severe clinical symptoms [10,83]. Addressing endoscopic POR proactively is critical, as its progression is closely associated with the development of significant postoperative complications.

### 3.5. Surgical Postoperative Recurrence

Before the introduction of biologic therapies, more than 50% of patients required a second ileocolonic resection within five years of their initial surgery [2,90]. Around 10% to 30% of individuals undergoing surgery for CD develop clinical recurrence within the first year following the procedure. Over the course of the first decade post-surgery, this figure rises to exceed 60%. Rates of surgical recurrence have been documented at approximately 20% to 25% within five years and between 34% and 49% at the ten-year mark [2,90]. A recent population-based study by Poulsen et al. covering 47.4% of the Danish population and including 631 CD patients who underwent primary resection between 2010 and 2020 reported contemporary re-resection rates lower than historical data [91]. Re-resection rates at 1, 5, and 10 years were 12.6%, 22.4%, and 32.2%, respectively, with 24.5% of patients requiring a second resection and 5.3% a third. When analyzing disease activity-driven re-resections specifically, rates were substantially lower at 3.6%, 10.1%, and 14.1% at 1, 5, and 10 years, respectively, as 40% of additional resections were performed for stoma reversal. The median time to recurrence was 11.0 months. Importantly, prophylactic biologic therapy initiated within 1 year of primary ileocecal resection demonstrated a protective effect against re-resection (HR 0.58, 95% CI 0.34–0.99, *p* = 0.047), particularly for stenotic and penetrating phenotypes. Risk factors for re-resection included location of resected bowel segments, disease location and behavior, smoking, and perianal disease. These findings suggest that contemporary re-resection rates may be declining with modern therapeutic strategies and that biologic therapy may be disease-modifying for certain subgroups when initiated early postoperatively.

A distinct clinical scenario exists for CD patients with permanent ileostomy, where postoperative management and recurrence assessment differ from those with intestinal continuity. When temporary or permanent stoma creation is required in CD patients, the postoperative course can be complicated by both conventional stoma-related issues and CD-specific complications [92]. In a prospective cohort of 54 consecutive CD patients undergoing stoma creation, complications occurred in 70% of cases at a median of 1.3 months postoperatively. Notably, 15% developed CD-related complications including pyoderma gangrenosum, peristomal fistulae, granulomas, and peristomal abscesses, with 20% requiring surgical revision. End-stoma configuration was significantly associated with higher rates of CD-related complications (*p* = 0.006), and patients with CD-related complications tended to have shorter disease duration (*p* = 0.07), suggesting that more aggressive disease phenotypes carry greater risk

A systematic review and meta-analysis by Abushamma et al. including 30 cohort studies with 2055 CD patients with permanent ileostomy (median age 32 years at ileostomy creation, most commonly performed for refractory disease) reported a pooled POR rate of 27% (95% CI 21.3–33.3) [93]. The study revealed significant heterogeneity in diagnostic approaches, with recurrence identified through symptoms (15 studies), endoscopy (4 studies), histology (2 studies), imaging (5 studies), and surgery (22 studies). These findings underscore the need for consensus guidelines specific to CD patients with permanent ileostomy, as current management strategies and trial endpoints are largely extrapolated from patients with intestinal continuity.

## 4. Risk Factors for Postoperative Recurrence After Ileocecal Resection

The challenge posed by POR highlights the need of identifying patients who are at greater risk. Several studies have investigated patient-related, disease-specific, surgical, and histological factors associated with POR in CD. A key element in postoperative management is the use of medical therapy to prevent recurrence. However, while risk stratification plays a vital role in guiding treatment decisions following intestinal resection, a validated risk assessment tool for predicting POR has yet to be developed [6].

Multiple clinical factors have been associated with an increased likelihood of POR. These include smoking, being under 30 years of age at the time of surgery, having a penetrating or perianal disease phenotype, small bowel involvement exceeding 50 cm, and a history of two or more prior surgeries for CD [5,6,94,95]. Among them, smoking is particularly notable as the only modifiable risk factor, underscoring the importance of perioperative counseling to encourage smoking cessation. Despite some overlap, different professional guidelines vary in their criteria for determining high-risk patients who should receive medical prophylaxis. The British Society of Gastroenterology in 2019 required at least two risk factors for classification as high-risk, but the latest guidelines have expanded this indication even to patients with one risk factor or with preference towards prophylaxis. Both the American Gastroenterological Association (AGA) and the European Crohn’s and Colitis Organization (ECCO) classify patients with even a single risk factor as high-risk [5,6,94,95].

The development of a standardized, validated risk assessment score would provide a much-needed framework for identifying which patients are most likely to benefit from early prophylactic interventions, enabling a more uniform and effective approach to postoperative management.

Risk factors linked to POR after ileocecal resection are represented in Table 2.

### 4.1. Clinical Characteristics

POR following ileocolic resection in CD is a complex phenomenon influenced by a multifaceted interplay of patient-related, disease-specific, and surgical factors.

As noted earlier, perhaps the most compelling patient-related risk factor is tobacco use [96]. Studies consistently demonstrate a significant association between smoking and an increased likelihood of both clinical and surgical recurrence [64,97,98]. This association is not merely correlative; evidence suggests that cessation of smoking can markedly reduce the risk of POR.

Moreover, the duration of CD prior to ileocecal resection emerges as a noteworthy consideration. While a shorter disease duration appears to increase the risk of POR, it is important to acknowledge the inherent challenges in defining a standardized timeframe across diverse patient populations [99,100,101]. Furthermore, the impact of age at disease onset and at the time of ileocecal resection on POR remains an area of active investigation, with existing research yielding conflicting results [5,94,95].

Recent evidence highlights preoperative body composition as a potentially modifiable risk factor for postoperative outcomes [102]. Bak et al. demonstrated in a prospective multicenter study of 227 CD patients undergoing ileocolic resection that myosteatosis, defined as high lipid content in skeletal muscle, was consistently associated with increased risk of overall postoperative complications (aOR 3.09, 95% CI 1.36–7.00), moderate-to-severe complications (aOR 2.66, 95% CI 1.24–5.68), and infectious complications (aOR 2.44, 95% CI 1.10–5.40) [103]. The study also revealed high lipid content in visceral adipose tissue as protective against endoscopic POR (aOR 0.26, 95% CI 0.07–0.99). Both low and high skeletal muscle index were paradoxically associated with increased risk of endoscopic POR, suggesting a U-shaped relationship.

Beyond body composition, dietary factors may influence postoperative outcomes. Bak et al. conducted a prospective multicenter study of 103 CD patients following ileocolic resection, analyzing 520 food diaries to assess nutrient intake associations with endoscopic POR [104]. Lower intake of specific micronutrients was associated with increased risk of endoscopic POR (modified Rutgeerts’ score ≥ i2a), including isoflavones such as genistein, daidzein, and glycitein, inositol, pinitol, provitamin-A carotenoid, xylitol, and parinaric acid.

Disease behavior also plays a significant role [105,106,107]. Patients with penetrating disease, or those exhibiting preoperative stricturing behavior, face an elevated risk of reoperation and the development of anastomotic strictures, which can lead to obstructive symptoms [108]. A recent international multicenter study by Avellaneda et al. involving 2013 patients who underwent ileocecal resection between 2012 and 2022 compared long-term outcomes between inflammatory (uncomplicated) and complicated CD phenotypes [109]. Despite complicated CD patients presenting with higher rates of preoperative anemia, emergent surgery, and open procedures, the study found comparable long-term endoscopic (HR: 1.03; *p* = 0.748), clinical (HR: 1.35; *p* = 0.073), and surgical recurrence rates (HR: 0.77; *p* = 0.419) between the two groups. Furthermore, the presence of perianal disease may signify a more aggressive CD phenotype, even in the absence of complex intra-abdominal disease manifestations, thereby warranting careful consideration in the overall treatment plan [106,110,111].

Finally, surgical history and disease location significantly influence the risk of POR. Prior surgical resections, particularly multiple resections, appear to increase the likelihood of future recurrence [63,106,110,112].

Emerging evidence suggests potential protective factors beyond traditional risk modification. A recent retrospective cohort study of 2421 patients with moderate-to-severe IBD found that statin users had significantly lower odds of IBD-related surgery compared to nonusers (5% vs. 9%, *p* = 0.007), with an adjusted hazard ratio of 0.47 (95% CI 0.26–0.85) independent of cardiovascular risk factors [113]. While not specifically studied in the postoperative setting, statins’ pleiotropic anti-inflammatory effects warrant investigation as potential adjunctive therapy for POR prevention.

### 4.2. Surgical Risk Factors

The surgical approach to ileocolic resection in CD significantly impacts the risk of POR. Avellaneda et al. confirmed the protective effect of laparoscopic surgery (HR: 0.74; *p* = 0.009) on recurrence rates [109].

Extensive resections, typically encompassing 20–50 cm of resected bowel have been identified as risk factors [110,114]. The association between ileocolonic anastomosis configuration and POR has been extensively investigated through 16 studies including four RCTs [97,106,108,112,115,116,117,118]. While side-to-side anastomoses are typically performed as anti-peristaltic totally stapled functional end-to-end configurations and end-to-end anastomoses as hand-sewn, results of side-to-side anastomosis remain controversial [119,120]. While, Canadian multicenter RCT randomizing 139 patients found no significant difference in endoscopic POR rates between end-to-end (42.5%) and side-to-side (37.9%) groups at 12 months (*p* = 0.55) [121]. ECCO guidelines suggest stapled side-to-side anastomoses for small-bowel or ileocolic resections in CD as an evidence level 3 recommendation [122]. Although, one RCT focusing on stapling technique demonstrated significant differences in surgical POR, with 9.1% in hand-sewn versus 28.8% in stapled ileocolonic anastomoses [123], the recommendation of performing side-to-side anastomosis represents the prevailing consensus, as this type of anastomosis is associated with lower rates of postoperative complications and allows for intracorporeal anastomosis [124,125]. Several meta-analyses support this approach, with one comparing 396 stapled side-to-side with 425 hand-sewn end-to-end anastomoses finding that stapled side-to-side outperformed across all endpoints including overall postoperative complications, anastomotic leak, recurrence, and reoperation for recurrence [126,127,128,129]. A network meta-analysis of 11 trials including 1113 patients further substantiated the superiority of stapled side-to-side anastomosis regarding overall complications, clinical recurrence, and reoperation for recurrence, though heterogeneity in POR definitions, follow-up length, and postoperative management limits interpretation [130].

Additionally, a recent Dutch study revealed that inverted stapled lines (longitudinal anastomosis) were frequently ulcerated in both CD patients and colorectal cancer controls, while everted stapled lines (closing blind loops) were not, suggesting these ulcerations result from secondary wound healing and ischemia induced by staple compression and may represent transient lesions that should not be overscored as endoscopic POR [131].

The mesentery plays a crucial role in the inflammatory milieu of CD. As discussed in Section 2.2, the mesentery contributes to a ‘pro-inflammatory’ environment through complex molecular interactions with the intestinal bowel wall. Consequently, strategies aimed at resecting or isolating the mesentery from the diseased area have been explored as potential avenues for reducing POR rates [22,132].

The evidence regarding extended mesenteric resection remains controversial, with significant heterogeneity across studies that warrants careful interpretation. Early retrospective studies suggested a potential benefit for more extensive mesenteric resections [133]. However, the SPICY trial, a randomized controlled study comparing mesenteric sparing to extended mesenterectomy in 118 patients, did not demonstrate a significant difference in endoscopic POR rates (Rutgeerts Score ≥ i2b) between groups (43% vs. 46%, *p* = 0.835) [134,135].

Several factors may explain the discrepant findings across studies. First, definitions of ‘extended’ versus ‘conventional’ mesenteric resection vary considerably, with no standardized surgical protocol. Second, patient populations differ in disease phenotype distribution; the SPICY trial enrolled predominantly inflammatory (B1) phenotypes, whereas retrospective series often included higher proportions of stricturing (B2) and penetrating (B3) disease where mesenteric involvement may be more relevant. Third, concurrent postoperative medical prophylaxis was not standardized across studies, potentially confounding the surgical effect. Fourth, timing of endoscopic assessment varied from 6 to 18 months, affecting recurrence detection rates.

A systematic review and meta-analysis by Mostafa and colleagues (2025) including 4358 patients across five studies found no significant differences between extended mesenteric resection and conventional mesenteric resection in disease recurrence (OR 0.89, 95% CI 0.67–1.18), re-operation rates, or postoperative morbidity [136]. Based on this evidence, we concur with current ECCO guidelines recommending a mesenteric-sparing approach as the standard of care.

Future research should address whether specific patient subgroups may benefit from extended mesenteric resection. Patients with significant mesenteric thickening or creeping fat on preoperative cross-sectional imaging may represent a population where mesenteric excision could provide additional benefits. Ongoing prospective trials are currently investigating the optimal role of mesenteric management and exploring potential synergistic effects with different anastomotic techniques [135,137]. The Kono-S anastomosis technique represents a distinctive approach that specifically excludes mesenteric tissue from the anastomotic area through a modified configuration. The procedure involves creating an antimesenteric, longitudinal incision that is then closed transversely, effectively separating the mesentery from the surgical anastomosis. This mesenteric exclusion has been hypothesized to reduce inflammatory triggers at the anastomotic site, given the recognized role of mesenteric tissue in perpetuating intestinal inflammation through complex molecular interactions [116]. A prospective trial (SuPREME-CD) comparing Kono-S to side-to-side anastomosis demonstrated decreased rates of endoscopic and surgical POR in the Kono-S group at three-year follow-up [138]. While a subsequent meta-analysis supported these findings, more recent studies, including a large nationwide propensity score-matched analysis and a recent large-scale multicenter study called the KoCoRICCO trial, have not consistently observed a significant difference in endoscopic POR rates between Kono-S and conventional anastomoses [139,140,141,142,143]. This highlights the ongoing debate regarding the optimal anastomotic technique for minimizing POR.

Several studies have investigated the association between postoperative complications and POR. Two retrospective studies have demonstrated an association between intra-abdominal septic complications, such as anastomotic leaks or abscesses, occurring within 90 days of ileocecal resection, and an increased risk of earlier POR [144,145]. If prospectively validated, this finding suggests that postoperative complications may represent an independent risk factor for POR, potentially exceeding the impact of preoperative clinical risk profiles. Moreover, a retrospective multicenter study by Carvello et al. found that the occurrence of postoperative complications is an independent risk factor for endoscopic POR after primary ileocecal resection for CD, affecting both the rate and timing of endoscopic and clinical recurrences [146]. Similarly, a previous retrospective study by Guo et al. observed a significantly higher risk of both clinical and surgical POR in patients experiencing postoperative abdominal septic complications [147]. While the precise mechanisms underlying this association remain elusive, it is hypothesized that a more aggressive CD phenotype may predispose patients to both increased POR rates and a higher risk of anastomotic complications related to impaired mucosal healing.

The existing literature on surgical techniques for the treatment of terminal ileitis in CD is characterized by a predominance of low-quality, retrospective studies with limited power. Thus, the results of these studies must be interpreted with caution. However, several ongoing RCTs are expected to provide more robust data on the impact of different anastomosis and resection techniques on the burden of POR [138].

A recent study has compared outcomes of strictureplasty as compared to those of ileocecal resection [148]. Strictureplasty represents a bowel-sparing surgical option for stricturing CD that avoids the loss of intestinal length. A recent propensity score-matched study of 42 patients who underwent strictureplasty demonstrated that intestinal inflammatory activity decreased even when diseased bowel was left in situ, as evidenced by reductions in fecal calprotectin levels at 12 months and improvements in MRI parameters including wall thickness, apparent diffusion coefficient, and validated activity scores (MaRIA and Clermont). However, surgical recurrence was significantly more frequent in the strictureplasty group compared to resection (*p* = 0.003), likely reflecting the fibrotic nature of strictures and the multifocal character of CD.

### 4.3. Histologic Risk Factors

Histological factors present at the time of ileocecal resection may offer insights into the likelihood of POR. Specific features, such as active disease at the resection margins, increased lymphatic vessel density, and mast cell infiltration within the subserosa, have been suggested as potential predictors of recurrence [149,150,151,152]. Additionally, metaplastic changes including Paneth cell metaplasia in the colon and pyloric metaplasia in the small bowel and right colon represent histological markers of chronic inflammatory stress that may indicate heightened recurrence risk. A meta-analysis of 21 studies involving 2236 patients highlighted the role of granulomatous disease in mesenteric lymph nodes, which was associated with both earlier and more frequent POR and subsequent reoperations [153]. Additionally, the presence of myenteric and submucosal plexitis has been linked to a heightened risk of endoscopic POR. Notably, the severity of myenteric plexitis correlates directly with the intensity of endoscopic POR, underscoring its relevance as a potential risk marker [149,154,155]. The constellation of these histopathological features at the time of resection may provide valuable prognostic information, though standardized assessment protocols and prospective validation remain necessary before routine clinical implementation.

The latest ECCO workshop emphasized that lymphatic vessel density warrants investigation, with one study on 28 CD patients demonstrating that patients with Rutgeerts score ≤ i1 at one year had higher lymphatic vessel density in both mucosa and submucosa of the proximal resection margin compared to those with score ≥ i3 [156]. Recent evidence suggests that while histological assessment of resection specimens provides valuable information, the combination of currently used histological risk factors with clinical factors brings only modest improvement in predicting endoscopic POR probability at 6 months compared to clinical factors alone (AUC 0.71 versus 0.70) [157].

### 4.4. Microbiome-Related Risk Factors

As discussed in Section 2.1, microbiota dysbiosis plays a central role in POR pathogenesis. From a clinical risk prediction perspective, several microbial signatures have demonstrated prognostic value. Reduced alpha diversity, characterized by an increase in Proteobacteria and a decrease in Firmicutes families such as *Lachnospiraceae* and *Ruminococcaceae*, has been associated with endoscopic POR [158]. At the time of surgical resection, *Ruminiclostridium* 6 was found to have a protective effect against endoscopic POR, while *Corynebacterium* was linked to an increased risk of recurrence. Notably, a study combining clinical risk factors with microbiota analysis demonstrated high predictive accuracy for 6-month endoscopic POR (AUC = 0.98), with cumulative risk increasing with additional factors [158]. Furthermore, adherent invasive *Escherichia coli* at the surgical site was associated with a 2.5-fold higher risk of 6-month endoscopic POR (OR 2.5, 95% CI 1.2–5.3) and was more frequently observed in patients with disease classified as ≥i1 [159].

A recent multicenter study by Hernández-Rocha et al. analyzing 349 postoperative colonoscopies from 262 patients demonstrated that microbiome deviations precede new-onset inflammation after surgically induced remission. Patients in remission at first colonoscopy who subsequently developed recurrence showed lower microbial diversity and specific taxonomic shifts. Microbiome features predicted future recurrence better than clinical risk factors alone, achieving an AUC of 0.98 when combined with clinical parameters [160].

These microbial signatures suggest that the intestinal microenvironment at the time of surgery and in the immediate postoperative period plays a crucial role in determining recurrence risk, though clinical application of microbiome-based risk stratification awaits further validation.

## 5. Postoperative Recurrence Management Strategies in CD

Based on the identified risk factors for POR, various professional societies have developed guidelines to categorize patients at high-risk [5,94,95]. These guidelines generally recommend the use of biologic prophylaxis as a preventive measure for individuals classified as high-risk. The societal recommendations are presented in Table 3.

Recent studies have highlighted the significant additive effect of multiple clinical risk factors on the likelihood of endoscopic POR [64,106]. The pivotal POCER trial by De Cruz et al. demonstrated the value of risk-based postoperative management combined with proactive surveillance [4]. In this trial, all participants received a 3-month course of metronidazole, while high-risk individuals were prescribed thiopurines or adalimumab if they were intolerant to thiopurines. Escalation to more aggressive therapies, such as weekly adalimumab, was initiated for patients with significant endoscopic recurrence (Rutgeerts score ≥ i2). Patients who underwent ileocolonoscopy at 6 months, followed by treatment adjustments based on mucosal findings, achieved an 18% reduction in endoscopic POR at 18 months compared to standard care.

The evolving role of surgery in CD management has been highlighted by the LIR!C trial, a multicenter RCT comparing early laparoscopic ileocecal resection to infliximab induction and maintenance therapy in patients with limited ileocecal CD (less than or equal to 40 cm of predominantly inflammatory disease) [162,163]. At one-year, laparoscopic resection demonstrated comparable quality of life outcomes to infliximab and was found to be cost-effective. Notably, at long-term follow-up with a median duration of 63.5 months, 42% of patients in the surgical group required no additional therapies and none necessitated reoperation, while 48% of the infliximab group subsequently required surgery. These findings support early surgical resection as a valid alternative to biologic therapy in carefully selected patients with limited, predominantly inflammatory ileocecal disease, challenging the traditional paradigm of reserving surgery only for medically refractory cases.

### 5.1. Systematic Medical Prophylaxis

**Figure 3:**
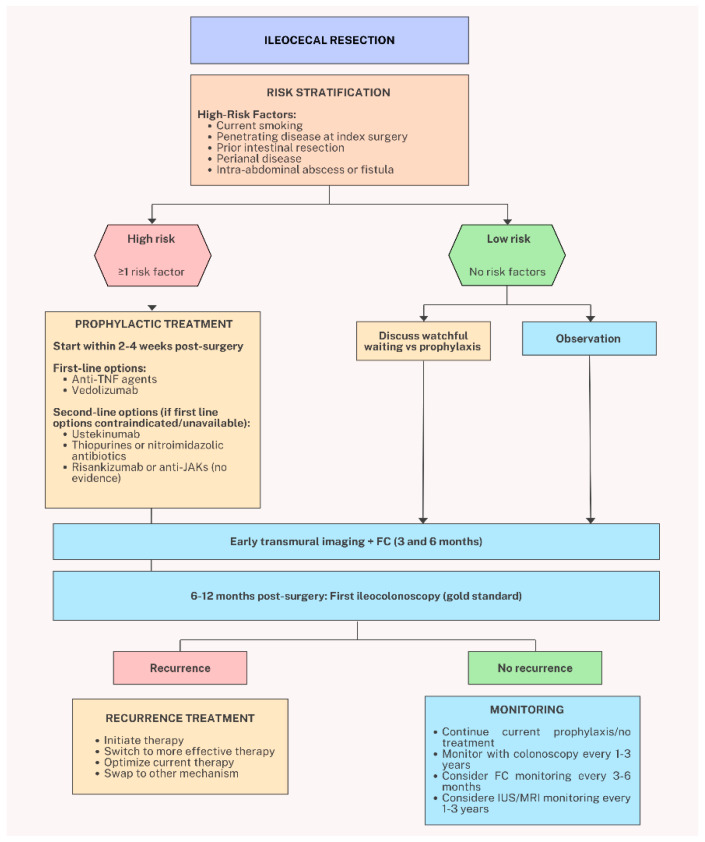
Algorithm for postoperative recurrence management in Crohn’s disease.

Systematic prophylaxis involves initiating pharmacologic therapy immediately post-surgery to pre-empt endoscopic recurrence. RCTs have shown that thiopurines and anti-TNFα agents effectively reduce endoscopic POR rates [164]. This approach is particularly beneficial for patients with prior complications or complex diseases, who are at higher risk of disease progression. However, it may lead to overtreatment in up to 20–30% of patients who would not develop endoscopic POR without therapy and in 40–50% of patients with low-risk lesions (Rutgeerts score i1–i2). The potential for drug-related side effects, such as those associated with imidazole antibiotics, further underscores the importance of balancing risks and benefits in this approach. A proposed algorithm for management of post-operative CD is illustrated in Figure 3.

### 5.2. Endoscopy-Driven Therapy

An alternative to systematic prophylaxis, endoscopy-driven strategies aim to avoid overtreatment by monitoring patients postoperatively (6–12 months) and initiating therapy only for those with severe lesions. Evidence suggests that thiopurines and anti-TNFα agents can resolve or improve mucosal lesions when introduced in this targeted manner [8,165]. However, studies such as Ferrante et al. found no significant differences between systematic prevention and endoscopy-driven treatment in terms of endoscopic POR at 18 months, though the study lacked statistical power [166]. Larger retrospective studies showed higher endoscopic POR rates within the first year for endoscopy-driven approaches compared to systematic prophylaxis [167]. Despite this, clinical POR rates at 3 years remained similar, supporting the use of endoscopy-driven strategies to minimize unnecessary treatments for low-risk individuals. However, subsequent real-world studies found higher endoscopic POR rates in the endoscopy-driven groups [168]. Despite these findings, endoscopy-driven therapy remains favored in some settings to avoid unnecessary treatment for low-risk patients. The modified Rutgeerts score distinguishes anastomotic lesions (i2a) from ileal lesions (i2b), with clinical implications. Anastomotic i2a lesions may partly reflect staple-line healing abnormalities rather than true recurrence and can sometimes resolve spontaneously. In contrast, i2b lesions represent true ileal recurrence requiring prompt therapeutic intervention. A pragmatic approach reserves treatment intensification for i2a persistence or progression while treating i2b lesions immediately.

### 5.3. Risk-Stratified Therapy

Risk-stratified approaches tailor treatment intensity to individual patient profiles. Key risk factors, such as smoking, prior resections, penetrating disease, and perianal involvement, are used to differentiate high- and low-risk groups [169]. High-risk patients are prioritized for systematic prophylaxis, as demonstrated in the REMIND study, which showed a direct relationship between the number of risk factors and the likelihood of endoscopic POR [170]. A Dutch cohort study confirmed that clinical risk stratification could reliably predict endoscopic recurrence (Rutgeerts score ≥ i2b) at 6 months, with limited additional benefit from histological parameters [171]. However, recent analyses have questioned the predictive value of some traditional risk factors.

Emerging biomarker-guided approaches offer the potential for more precise individualization of prophylactic therapy. Fecal calprotectin has demonstrated utility in identifying patients at high risk for endoscopic relapse or POR, and serial monitoring of calprotectin kinetics may guide treatment intensification decisions [172]. Serological markers reflecting immune responses to gut microbiota and autoantigens, including anti-Saccharomyces cerevisiae antibody (ASCA), antibody to *Escherichia coli* outer membrane porin C (OmpC), anti-flagellin antibody (CBir1), and granulocyte-macrophage colony-stimulating factor autoantibodies, have been associated with disease course and complicated phenotypes in CD, with positivity for multiple antimicrobial antigens correlating with faster progression to complicated disease [173]. Microbiome signatures represent another promising avenue for personalized risk assessment, with studies demonstrating that specific taxa, including reduced abundance of *Faecalibacterium* and *Lactobacillus* and increased *Ruminococcus gnavus* and *Gammaproteobacteria*, are associated with endoscopic POR independent of anti-TNF use [174]. Transcriptomic profiling of ileal tissue at the time of surgery has identified distinct whole-transcriptome signatures in patients with indolent versus aggressive disease courses, and serum proteomic panels such as the endoscopic healing index are under investigation for their ability to predict mucosal healing and guide treatment decisions [175]. Machine learning-based prognostic models integrating clinical, serological, genetic, and molecular data are being developed, with some models demonstrating good discrimination for postoperative recurrence prediction [176,177]. However, prospective validation of these emerging biomarker panels remains essential before their integration into routine clinical practice, and ongoing research aims to establish validated algorithms that combine multiple biomarker modalities for truly personalized postoperative management.

Several studies have evaluated the efficacy of prophylaxis versus endoscopy-driven strategies. Joustra et al. demonstrated a significant reduction in 12-month endoscopic POR (Rutgeerts score ≥ i2b) in high-risk patients receiving prophylactic biologics compared to endoscopy-driven treatment (24.3% vs. 44.5%, *p* < 0.05) [168]. However, no differences in clinical POR were observed at 36 months. Arkenbosch et al. found lower endoscopic POR rates in both high-risk (26% vs. 49%) and low-risk (16% vs. 45%) groups receiving prophylaxis, although adherence to prophylactic recommendations was suboptimal in high-risk patients [171]. Shah et al. found reduced surgical recurrence rates (10.2% vs. 16.7%, *p* = 0.02) and lower endoscopic POR across all risk levels in patients receiving biologic prophylaxis [178]. Table 4 gives an overview of studies published regarding the management of POR.

### 5.4. Long-Term Considerations and Ongoing Trials

While early biologic prophylaxis has demonstrated benefits in reducing endoscopic POR, its impact on long-term outcomes remains unclear. Studies assessing late endoscopic POR risk have shown recurrence rates of 40–50% even in patients with initial postoperative remission, particularly those with baseline Rutgeerts scores of i1 or i2a [183,184]. Regular monitoring, such as colonoscopy within the first postoperative year, has been associated with a 69% reduction in surgical recurrence risk (HR 0.31, *p* = 0.005) [185]. Ongoing studies, including the SOPRANO-CD trial (NCT05169593, https://clinicaltrials.gov/study/NCT05169593, accessed on 24 November 2025), are expected to further refine postoperative management strategies by comparing systematic biologic prophylaxis with endoscopy-driven approaches [185].

The SOPRANO-CD trial is a Phase 4 RCT comparing systematic biological therapy (adalimumab, infliximab, ustekinumab, vedolizumab, or risankizumab) started within 14 to 40 days post-surgery versus endoscopy-driven therapy initiated only at week 30 if endoscopic recurrence develops in CD patients after ileocolonic resection. The study enrolled patients aged 18–80 with at least one risk factor for POR (penetrating disease, active smoking, previous resections, but also considers recent biological therapy) who previously failed steroids or immunosuppressives, measuring primary outcomes of endoscopic recurrence at week 86 and need for unscheduled treatment adaptation, with secondary outcomes including clinical recurrence, quality of life, costs, and adverse events. Recruitment began on September 2022 with estimated primary completion in October 2027 and final completion on October 2030, and the trial is still actively recruiting across 28 sites in Belgium and Italy.

These trials will provide valuable insights into the balance between preventing recurrence and avoiding overtreatment, paving the way for personalized care in CD. Biosimilars represent an important advancement in the long-term postoperative management of CD by improving accessibility and cost-effectiveness of biologic prophylaxis. Since the approval of the first infliximab biosimilar (CT-P13) in 2013, multiple biosimilars for both infliximab and adalimumab have entered clinical practice [186,187]. Meta-analyses and real-world observational studies involving thousands of patients have demonstrated that biosimilars maintain comparable efficacy, safety, and immunogenicity profiles to their originator compounds in inflammatory bowel disease. Switching from reference infliximab or adalimumab to biosimilars has been shown to be effective and safe, with no significant loss of efficacy or increased immunogenicity, allowing cost savings to be reinvested in earlier treatment initiation and expanded patient access to biologic therapy. The introduction of biosimilars has substantially increased biologic utilization in European countries, with infliximab use increasing by approximately 90% since biosimilar market entry.

Advanced drug delivery systems offer additional opportunities to improve treatment adherence and outcomes in long-term postoperative maintenance [188]. The recent approval of subcutaneous infliximab (CT-P13 SC) represents a significant advancement, providing patients with a convenient alternative to intravenous infusions that require hospital visits [189]. The LIBERTY-CD and LIBERTY-UC phase III trials demonstrated superiority of subcutaneous infliximab 120 mg every two weeks over placebo in achieving clinical remission and endoscopic response following intravenous induction therapy. Subcutaneous formulations offer several advantages including patient convenience through at-home self-administration, stable and consistently higher serum drug levels, reduced immunogenicity with lower anti-drug antibody formation, and decreased healthcare resource utilization. This formulation may be particularly beneficial in refractory patients, with studies suggesting that switching from intravenous to subcutaneous infliximab can achieve therapeutic drug levels even in patients with prior inadequate response [190]. Looking toward the future, nanoparticle-based drug delivery systems are being developed to target the inflamed intestine directly, with the potential to increase local drug concentrations at disease sites while minimizing systemic exposure and side effects [191]. Multi-responsive nanocarriers that release drugs in response to pH, reactive oxygen species, or enzymatic triggers specific to the inflammatory bowel disease microenvironment are under investigation and may eventually offer more precise and personalized therapeutic approaches in the postoperative setting. Management of POR requires consideration of specific patient characteristics and recurrence patterns. Patients with preoperative biologic failure present a clinical dilemma; however, available evidence suggests anti-TNF agents may retain efficacy. Patients with primary non-response to multiple anti-TNF agents may benefit from switching to vedolizumab or ustekinumab. Concomitant perianal disease signifies a more aggressive phenotype warranting intensified prophylaxis and close surveillance regardless of other risk factors. Data on elderly and pediatric populations remain limited; in elderly patients, infection risk and comorbidities require careful consideration, while in pediatric patients, growth concerns support aggressive disease control with early biologic prophylaxis in high-risk cases.

## 6. Postoperative Non-Invasive Monitoring

Data from the POCER trial showed that early endoscopic evaluation at 6 months postoperatively, coupled with medication adjustments, significantly reduced endoscopic POR rates. Consequently, most guidelines recommend ileocolonoscopy within 6–12 months after surgery [4]. Despite these recommendations, real-world studies highlight suboptimal adherence to timely endoscopic monitoring within this period [192]. Proposed strategies now include non-invasive diagnostics to assess POR to reduce invasiveness, cost, and patient burden have spurred interest in alternative modalities, offering risk-stratified biomarker assessments [193]. However, no universally optimal monitoring strategy has yet been established.

### 6.1. Biomarkers

FC, a reliable neutrophilic protein biomarker, has been extensively studied for monitoring CD recurrence [194,195]. A meta-analysis evaluating 613 patients found that FC demonstrated a pooled sensitivity of 0.82 (95% Confidence interval [CI]: 0.73–0.89) and specificity of 0.61 (95% CI: 0.51–0.71) for detecting endoscopic recurrence, with recurrence defined as a Rutgeerts score ≥ i2. Another meta-analysis showed similar results, identifying the optimal diagnostic range for FC as 100–150 μg/g. Interestingly, when using a modified Rutgeerts score, FC showed improved sensitivity and specificity compared to the traditional scoring method, especially in differentiating between i2a (anastomotic) and i2b (ileal) lesions [196].

The AGA recently proposed a FC cutoff of <50 μg/g for patients with a low pre-test probability of recurrence to avoid routine endoscopy [62]. This threshold was also recommended for individuals on postoperative prophylaxis with one or more risk factors for recurrence. For asymptomatic patients at low risk and receiving prophylactic treatment, an FC level < 150 μg/g may negate the need for routine endoscopic assessment during the first postoperative year. However, the utility of continued FC monitoring beyond the first year is yet to be determined.

Combining FC with serum biomarkers has emerged as a promising predictive tool. A study involving 61 patients measured cytokines, including IL-6 and IFN-γ, alongside FC at intervals before and after surgery. At 6 months postoperatively, FC ≥ 260 μg/g correlated strongly with POR. The combination of FC, IL-6, and IFN-γ achieved an AUC of 0.90, indicating high predictive accuracy for early recurrence [197,198].

Although C-reactive protein (CRP) is commonly used to assess inflammation, its sensitivity for intestinal inflammation in CD is limited. In the perioperative period, CRP monitoring showed a sensitivity of ~30% and specificity of ~90% [62]. Elevated CRP levels, particularly in high-risk individuals, can suggest recurrence and warrant confirmatory ileocolonoscopy.

Recent innovations include the Endoscopic Healing Index (EHI), which demonstrated similar accuracy to FC in detecting endoscopic POR at 6 months. An EHI < 20 showed a negative predictive value of 76% and sensitivity of 70% for endoscopic POR, which improved when combined with FC < 100 μg/g, yielding a 92% negative predictive value [199]. Emerging “-omic” biomarkers, such as CXCL9, have been linked to higher predictive accuracy for endoscopic POR, particularly when paired with CRP [200]. Urinary markers, like levoglucosan, have also shown potential for early POR detection, though these findings remain under investigation [201].

### 6.2. Radiologic Monitoring

Non-invasive radiologic techniques, including IUS, computed tomography enterography (CTE), and magnetic resonance imaging (MRI), have proven effective for postoperative monitoring [202,203]. Transmural inflammation characteristic of CD often affects bowel wall regions beyond the reach of endoscopic assessment. Non-invasive imaging modalities can address these limitations, offering valuable insights into disease activity while sparing patients the invasiveness of endoscopy.

#### 6.2.1. Intestinal Ultrasound

The utility of IUS in identifying POR of CD was first recognized in 1986, with numerous subsequent studies validating its efficacy [204,205,206,207,208]. IUS offers a quick, non-invasive, point-of-care method to assess bowel wall thickness, bowel wall vascularity, mesenteric fat hypertrophy, and lymph node enlargement, with these findings serving as indicators of active inflammatory disease [209,210,211]. The technique’s real-time capability enables assessment of functional consequences of strictures, including direct visualization of swirling intraluminal contents proximal to narrowed segments—an intuitive marker of obstructive physiology. Technological advancements in ultrasonography have facilitated the use of several specialized techniques, such as small intestine contrast or contrast-enhanced IUS. Two meta-analyses evaluating POR defined as bowel wall thickness ≥3 mm, had a pooled sensitivity and specificity values of 83.5% and 91.5%, respectively, when compared to endoscopic POR defined as Rutgeerts’ score ≥ i1 [204,212]. Moreover, a bowel wall thickness ≥ 5.5 mm is strongly associated with severe endoscopic POR, corresponding to Rutgeerts’ score ≥ i3 [204] When combining a bowel wall thickness of ≥3 mm and lymphadenopathy on ultrasound with FC levels ≥ 50 mcg/g, the positive predictive value for endoscopic POR reached 100%, while the absence of all three findings yielded a negative predictive value of 83% [213].

Among different non-invasive methods for monitoring POR, IUS has been shown to exhibit greater specificity than FC at 3 months post-surgery in at least one study [214].

Further research into contrast-enhanced IUS explored its ability to grade recurrence severity, with severe recurrence defined as a Rutgeerts score of ≥i3. The addition of intravenous contrast improved diagnostic accuracy, particularly when wall thickness was ≥6 mm or 5–6 mm with ≥70% bowel wall contrast enhancement or extra-intestinal complications. This approach achieved sensitivity of 90.3%, specificity of 87%, and an accuracy of 88.9% [208]. A recent small-scale study reported stronger correlations between intestinal US findings and endoscopic recurrence compared to biomarkers [205]. Early postoperative IUS findings, including increased bowel wall thickness (≥3 mm), hyperemia, and mesenteric lymphadenopathy, are associated with high likelihood of endoscopic recurrence at 12 months, enabling timely medical intervention and potentially altering the need or timing of endoscopic confirmation. Combined with FC, IUS enhances predictive value significantly: a BWT ≥ 3 mm and FC ≥ 50 mg/g predicts POR in up to 75% of cases, while values below these thresholds identify non-recurrence with 74% accuracy [54]. In patients with bowel-sparing procedures like strictureplasty, BWT > 6 mm at 6 months suggests high recurrence risk and should prompt therapy escalation or surgical reassessment [215]. According to the latest ECCO guidelines, bowel wall thickness ≥ 5.5 mm at 6 months postoperatively strongly indicates severe endoscopic recurrence and can be used to initiate therapy [202,203]. Furthermore, the ECCO consensus recommends an initial assessment at 3 months after surgery or treatment withdrawal; if findings are negative at 3 months, monitoring can be extended within 12 months, whereas positive findings at early evaluation may warrant earlier therapeutic intervention. IUS can also identify subtle postoperative complications such as anastomotic leaks or fluid collections before clinical deterioration, often reducing reliance on CT or MRE [216,217]. The technique’s repeatability and accessibility make it particularly valuable for personalized postoperative monitoring strategies, where high-risk patients may require closer surveillance while low-risk individuals can be managed with less intensive approaches.

While US shows high diagnostic performance, broader validation of scoring systems and larger studies are essential for widespread clinical application [218,219,220]. CTE, MRE, and video capsule endoscopy, as compared to IUS, also demonstrate high sensitivity and adequate specificity for detecting POR [59,212,221,222].

#### 6.2.2. Cross-Sectional Imaging

MRI has emerged as a reliable tool for detecting postoperative CD recurrence, with pooled sensitivity, specificity, and accuracy of 97% (95% CI, 0.89–1.00), 84% (95% CI, 0.62–0.96), and an AUC of 0.98, as reported in a recent systematic review and meta-analysis [212]. The MONITOR index, a scoring system tailored for POR detection via MRE, evaluates seven key radiographic features, including bowel wall thickness, contrast enhancement, and edema. A threshold score of ≥1 yields sensitivity and specificity of 79% and 55%, respectively, with the Area Under the Receiver Operating Characteristics (AUROC) increasing to 0.85 following validation. Despite its potential, the low negative predictive value necessitates further refinement and validation [212].

CTE has also demonstrated strong diagnostic accuracy, with sensitivity and specificity of 92.3% and 83.3% for identifying anastomotic recurrence [221,223]. Although less commonly utilized in routine practice, CTE has proven valuable in distinguishing between disease recurrence and fibrostenosis of the ileocolonic anastomosis, with stratification and the comb sign identified as the most discriminating features [224].

Notably, imaging can identify patients with transmural disease activity in the absence of luminal endoscopic POR, a condition linked to an elevated risk of disease progression highlighting the potential for non-invasive, multimodal strategies to become integral components of future monitoring protocols [59]. Although endoscopy provides the advantage of direct mucosal visualization, it does not comprehensively evaluate the intestinal wall or vasculature.

## 7. Medical Prophylaxis

Given the high likelihood of POR following ileocolic resection for CD, proactive treatment strategies are essential. Medical prophylaxis initiated shortly after ICR has shown promise in delaying or preventing POR. Various non-biologic agents, such as 5-ASA, antibiotics and immunomodulators, have been studied in this context [8,85,225,226,227,228,229]. 5-ASA and purine analogs are not currently suggested for post-surgical maintenance of remission of CD in the latest British guidelines [161]. Recent population-based data from Denmark further supports the protective role of early postoperative biologic therapy, demonstrating that biologics initiated within 1 year of resection reduced both disease recurrence and re-resection risk (HR 0.58, 95% CI 0.34–0.99, *p* = 0.047), with particular benefit observed in stenotic and penetrating disease phenotypes [91]. According to the latest BSG guidelines, anti-TNF therapy (infliximab or adalimumab) or vedolizumab should be considered after ileocolonic resection in CD patients with significant risk factors for recurrence, endoscopic evidence of disease at 6 months post-surgery, or when patients prefer early treatment through shared decision-making [161].

In the latest ECCO consensus, nitroimidazolic antibiotics (metronidazole and ornidazole) were considered to be effective in reducing endoscopic POR when used short-term (3 months for metronidazole, 1 year for ornidazole) [8]. However, due to the high rates of adverse the consensus did not generally recommend these drugs for long-term use.

The 2019 Cochrane network meta-analysis was updated by the British Society of Gastroenterology in 2025, incorporating 35 trials with 3249 participants and newly available vedolizumab data from the REPREVIO trial [161]. The updated analysis found with low certainty that adalimumab may have a large effect in preventing clinical and endoscopic relapses (HR 0.1; 95% CI 0.02–0.33 for clinical relapse and HR 0.1; 95% CI 0.01–0.32 for endoscopic relapse), while infliximab may have a moderate effect (HR 0.36; 95% CI 0.02–1.74 for clinical relapse and HR 0.24; 95% CI 0.01–1.2 for endoscopic relapse). Most notably, vedolizumab demonstrated with moderate certainty a probable large effect in preventing endoscopic relapse, representing higher certainty than achieved for anti-TNF agents.

Beelen et al. pooled six trials comparing anti-TNF therapy with thiopurines in 425 patients [230]. Anti-TNF therapy showed superiority for endoscopic recurrence (RR 0.52; 95% CI 0.33–0.80), clinical recurrence (RR 0.50; 95% CI 0.26–0.96), and severe endoscopic recurrence (RR 0.41; 95% CI 0.21–0.79). These findings remained robust in patients with prior anti-TNF exposure. Altogether, these analyses support biologic therapy, particularly anti-TNF agents and vedolizumab, as the most effective prophylactic strategy and confirm minimal benefit from mesalamine and modest but inferior efficacy of thiopurines.

Network meta-analyses have compared prophylactic strategies for preventing POR in CD. Singh et al. in 2015 analyzed 26 RCTs with 2203 patients, comparing mesalamine, antibiotics, thiopurines and anti-TNF agents [231]. Anti-TNF therapy demonstrated superiority over both placebo (OR 0.14; 95% CI 0.03–0.79) and thiopurines (OR 0.10; 95% CI 0.01–0.81) for preventing endoscopic POR, while mesalamine and antibiotics showed no significant benefit over placebo. More recently, Hupé et al. published a network meta-analysis in 2025 including 20 studies with 2414 patients, incorporating data on ustekinumab and vedolizumab [232]. The analysis confirmed that ustekinumab (OR 0.23; 95% CI 0.07–0.70), vedolizumab (OR 0.17; 95% CI 0.05–0.59), infliximab (OR 0.18; 95% CI 0.36–0.88), and adalimumab (OR 0.17; 95% CI 0.07–0.42) were all superior to placebo for preventing endoscopic POR, while thiopurines and 5-ASA showed no significant benefit. Based on SUCRA rankings, adalimumab (0.81), infliximab (0.80), vedolizumab (0.79), and ustekinumab (0.72) demonstrated the highest likelihood of being the most effective agents. No significant differences were observed between the four biologics. A comprehensive 2025 network meta-analysis by Gordon et al. including 34 RCTs (*n* = 3197) provided GRADE-assessed evidence for postoperative prophylaxis [233]. For clinical relapse prevention, adalimumab demonstrated moderate certainty evidence with a large effect size (RR 0.31, 95% CI 0.16–0.60, NNT = 3), while 5-ASA and purine analogs showed only trivial effects (RR 0.79 for both) with low certainty. For endoscopic relapse, vedolizumab achieved moderate certainty evidence (RR 0.37, 95% CI 0.17–0.80, NNT = 2, large effect), followed by adalimumab with low certainty (RR 0.47, 95% CI 0.27–0.80, NNT = 3). Notably, infliximab evidence was rated very low certainty, though this should not be interpreted as evidence of no effect. These findings reinforce current guideline recommendations favoring advanced therapies for high-risk patients. An important barrier to advancing postoperative management is the heterogeneity of endpoints across clinical trials. A systematic review by the IOIBD identified 42 RCTs assessing postoperative management, with highly variable definitions of recurrence, timing of assessment (3–24 months), and endoscopic thresholds (>i1 vs. >i2). The IOIBD consensus recommended that endoscopy represents the principal short-term endpoint for clinical trials, with endoscopic recurrence reproducibly defined and validated for prognostic relevance. Longer-term endpoints should incorporate evidence of macroscopic inflammation by imaging or endoscopy, or the presence of penetrating or stricturing complications. Regulatory agencies currently recommend clinical evaluations as for luminal CD, which may not be appropriate given the poor correlation between symptoms and endoscopic findings in the postoperative setting [74].

### 7.1. Anti-Tumor Necrosis Factor (Anti-TNFα) Agents

The efficacy of anti-TNFα agents in preventing POR was first demonstrated by Regueiro et al. in a small randomized controlled trial of 24 patients who received infliximab or placebo within 4 weeks of ileocolonic resection [192]. In this pilot study infliximab significantly reduced endoscopic POR at 1 year (9.1% vs. 84.6%, *p* = 0.0006), providing the initial proof of concept for anti-TNFα therapy in the postoperative setting. Subsequently, the PREVENT trial, a larger multicenter RCT, confirmed these findings [9]. This study randomized patients within 45 days of ICR to receive infliximab or placebo every 8 weeks for 200 weeks [9]. The infliximab group experienced a significantly lower rate of endoscopic POR (22.4% vs. 51.3%) compared to placebo. Although infliximab also reduced rates of clinical recurrence, the difference was not statistically significant.

The first evidence of adalimumab’s efficacy in preventing postoperative CD recurrence was demonstrated by Savarino et al. in a randomized controlled trial comparing adalimumab to azathioprine and mesalamine in 51 patients after ileocolonic resection [165]. Adalimumab showed significantly lower rates of both endoscopic POR (6.3% vs. 64.7% with azathioprine and 83.3% with mesalamine) and clinical recurrence (12.5% vs. 64.7% with azathioprine and 50% with mesalamine) at 2 years. Additional evidence from prospective pilot studies and a subgroup analysis from the POCER trial further confirmed that adalimumab significantly reduced rates of endoscopic recurrence compared to thiopurines [4,165,234]. A recent meta-analysis confirmed that anti-TNFα agents, including infliximab and adalimumab, outperformed other therapies in reducing endoscopic POR (≥i2), clinical recurrence (CDAI ≥ 300, HBI ≥ 8), and severe POR (≥i3) [230,235,236,237]. Notably, no significant differences in efficacy were observed between infliximab and adalimumab for either endoscopic or clinical POR.

**Figure 4:**
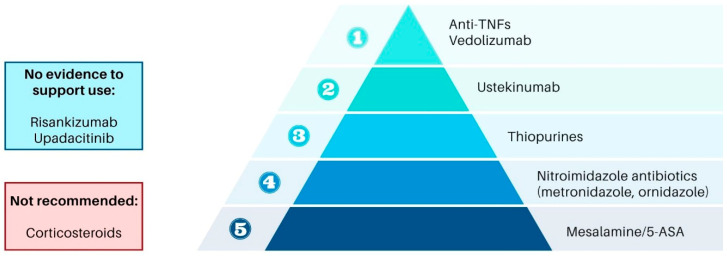
Pharmacological prophylaxis for postoperative recurrence: hierarchy based on efficacy and guideline recommendations.

Based on the cumulative evidence from network meta-analyses, RCTs, and current guideline recommendations, a hierarchical approach to pharmacological prophylaxis can be proposed (Figure 4).

The potential impact of prior anti-TNFα treatment failure on the effectiveness of these agents in preventing POR has been debated. In a retrospective study of 57 postoperative patients treated with anti-TNFα therapy initiated within 3 months of surgery, prior exposure to multiple anti-TNFα agents (≥2) was identified as a risk factor for both clinical and endoscopic recurrence [238]. Patients exposed to ≥2 anti-TNFα agents had a higher cumulative rate of recurrence at 2 years (45.5%) compared to those exposed to ≤1 agent (29.1%, *p* = 0.07). Multivariable analysis further corroborated this, with prior exposure to ≥2 agents associated with a higher risk of recurrence (HR = 4.2; 95% CI: 1.8–10.2; *p* = 0.001).

A recent retrospective multicenter study suggested that anti-TNFα therapy remains effective for POR prophylaxis in patients with preoperative anti-TNFα failure. Among 119 patients, those receiving anti-TNFα therapy (*n* = 71) had lower recurrence rates at 2 years (23.9%) compared to those on alternative treatments, including ustekinumab or vedolizumab (44.9%, *p* = 0.011) [239]. Furthermore, a subgroup analysis from an individual participant data meta-analysis of 6 RCT reaffirmed the superiority of anti-TNFα agents over azathioprine in patients previously exposed to anti-TNFα agents [230].

### 7.2. Combination Therapy

The combination of anti-TNFα agents with immunomodulators has shown increased efficacy compared to monotherapy [240]. Retrospective analyses indicate that combination therapy can reduce the risk of endoscopic POR by more than twofold in patients with prior anti-TNFα failure. Despite these promising findings, prospective studies are needed to determine the benefits of combination therapy in anti-TNFα-naïve populations [240].

### 7.3. Vedolizumab

Vedolizumab has shown potential as a prophylactic treatment for POR. Retrospective multicenter studies demonstrated successful POR prevention in 66–73% of cases [241,242]. The REPREVIO trial is the first prospective, multicenter RCT evaluating vedolizumab in the prevention of endoscopic POR after ICR [243]. Patients who underwent ICR and had ≥1 risk factor (active smoking, prior ICR, surgery for a perforating complication, previous exposure to anti-TNFα) were randomized to receive vedolizumab (*n* = 43) or placebo (*n* = 37) at week 0, 8, 16, and 24 after surgery. Nearly half (49%) of patients were Anti-TNFα exposed. Publicly presented data from the trial shows that patients on vedolizumab had a greater chance of endoscopic remission (77% vedolizumab vs. 38% placebo, *p* = 0.0004) and having a lower Rutgeerts score than those on placebo, despite similar rates of clinical recurrence.

### 7.4. Ustekinumab

Ustekinumab has emerged as a promising option for POR prevention. Retrospective studies comparing ustekinumab to azathioprine found lower endoscopic POR rates in the ustekinumab group (28% vs. 54.5%; *p* = 0.029) [244]. Additionally, comparisons with adalimumab revealed similar efficacy in reducing POR, even in patients with prior biologic failure [244]. Data from the ENEIDA registry support the use of ustekinumab in high-risk cohorts, where 42% of patients treated with ustekinumab experienced endoscopic POR within 18 months [245].

### 7.5. Emerging Therapies

Despite the expanding of advanced therapies, currently no studies have evaluated the use of Janus kinase inhibitors or Risankizumab for POR prevention [246,247]. These agents, with their unique mechanisms of action, warrant further investigation in the postoperative setting. Current evidence on drugs used in the prevention of POR are illustrated in Table 5.

## 8. Treatment of Postoperative Recurrence

The investigation into advanced therapies for managing endoscopic or clinical POR in CD remains limited. Emerging evidence from pilot studies suggests infliximab and adalimumab might offer superior endoscopic healing compared to conventional therapies [235,258]. Additionally, a post hoc analysis of the POCER trial indicated that adalimumab therapy following endoscopic detection of POR yielded higher healing rates than thiopurine treatment [4]. Furthermore, a retrospective study involving 44 patients with baseline endoscopic POR (observed at 6–12 months post-surgery) reported that ustekinumab treatment resulted in endoscopic healing (≥1-point reduction in Rutgeerts’ score) in 50% of patients, with 27.3% achieving scores of i0 or i1 [259].

The management of POR despite ongoing prophylactic therapy represents a common clinical challenge. Several strategic options exist, including dose optimization of the current agent, addition of an immunomodulator, or switching to a different biologic class. The choice between these approaches should be guided by therapeutic drug monitoring, the severity of recurrence, and prior treatment history. For patients with subtherapeutic drug levels or detectable anti-drug antibodies, dose optimization or addition of an immunomodulator may restore therapeutic efficacy. However, for patients with adequate drug levels who nonetheless develop POR, switching to a different mechanism of action appears more effective. Evidence supports this approach: among 81 patients with endoscopic POR while on biologic prophylaxis, those who switched biologic class achieved higher rates of endoscopic remission (52%) compared to those who underwent dose optimization alone (28%, *p* = 0.03) [260]. The postoperative setting presents unique considerations compared to luminal disease management in non-surgical patients. First, the altered intestinal anatomy may affect drug pharmacokinetics, particularly for oral agents. Second, the pattern of recurrence may inform treatment selection: anastomotic-confined lesions (i2a) may reflect local healing abnormalities and respond differently than true ileal recurrence (i2b), which suggests systemic disease activity requiring mechanism switching. Third, patients with POR despite anti-TNF prophylaxis may benefit from switching to vedolizumab or ustekinumab, given their gut-selective mechanisms and favorable safety profiles for long-term maintenance. A treat-to-target approach remains essential, with endoscopic re-evaluation recommended 6–12 months after treatment modification to assess response and guide further management decisions. Serial fecal calprotectin monitoring can provide interim assessment of treatment response, with persistently elevated levels (>150 μg/g) warranting earlier endoscopic evaluation.

Table 6 summarizes studies on the efficacy of POR therapies.

## 9. Conclusions

POR remains a major challenge in CD management despite advances in both surgical techniques and medical therapies. Although anti-TNF agents have demonstrated robust efficacy and newer biologics showed promise, the optimal balance between prophylactic treatment and endoscopy-driven intervention continues to evolve through risk stratification and personalized approaches. Future advancement will require integrating clinical, histological, genetic, and microbial data to refine treatment algorithms and prevent recurrence while minimizing unnecessary therapeutic exposure.

## Figures and Tables

**Table 1:** Endoscopic scoring systems for postoperative recurrence in patients with Crohn’s disease undergoing ileocecal resection.

Score	Description of Mucosal Findings
**Rutgeerts Score** [10]	
**i0**	No visible lesions in the distal ileum.
**i1**	≤5 small aphthous lesions in the distal ileum.
**i2**	>5 aphthous lesions with normal mucosa in between; may include skipped areas or lesions confined to the ileocolonic anastomosis.
**i3**	Extensive aphthous ileitis with widespread mucosal inflammation.
**i4**	Severe diffuse inflammation with large ulcers, nodules, or stenosis in the neo-terminal ileum.
**Modified Rutgeerts Score** [55]	
**i0**	Absence of lesions in the distal ileum.
**i1**	≤5 aphthous lesions in the distal ileum.
**i2a**	Lesions confined to the ileocolonic anastomosis, with or without <5 aphthous lesions in the ileum.
**i2b**	>5 aphthous lesions in the ileum with intervening normal mucosa; may include associated anastomotic lesions.
**i3**	Extensive aphthous ileitis with diffuse mucosal inflammation.
**i4**	Severe diffuse inflammation with large ulcers, nodules, or stenosis in the neo-terminal ileum.
**Proposed Updated Rutgeerts Score** [77]	
**i0**	No visible abnormalities in the neo-terminal ileum, anastomotic line, ileal inlet, or ileal body.
**i1**	≤5 aphthous lesions in the neo-terminal ileum, ileal inlet, or ileal body, with normal mucosa in between.
**i2a**	Lesions confined to the ileocolic anastomotic line, with or without <5 aphthous lesions in the neo-terminal ileum, ileal inlet, or ileal body.
**i2b**	>5 aphthous lesions with intervening normal mucosa or skip areas of larger ulcers in the neo-terminal ileum, ileal inlet, or ileal body, with or without anastomotic lesions.
**i3**	Extensive aphthous ileitis with widespread mucosal inflammation in the neo-terminal ileum, ileal inlet, or ileal body.
**i4**	Severe diffuse inflammation with large ulcers, nodules, or stenosis in the neo-terminal ileum, ileal inlet, or ileal body.
**REMIND Score** [78]	
**Anastomotic Lesions (<1 cm after anastomosis)**	
**A (0)**	No anastomotic lesion.
**A (1)**	Ulceration involving < 50% of the anastomosis circumference.
**A (2)**	Ulceration involving > 50% of the anastomosis circumference.
**A (3)**	Presence of anastomotic stenosis.
**Ileal Lesions**	
**I (0)**	No visible ileal lesion.
**I (1)**	≤5 aphthous lesions.
**I (2)**	>5 aphthous lesions with intervening normal mucosa or skip areas of larger lesions.
**I (3)**	Extensive aphthous ileitis with diffuse mucosal inflammation.
**I (4)**	Severe diffuse inflammation with large ulcers, nodules, or stenosis in the neo-terminal ileum.

**Table 2:** Risk factors potentially contributing to postoperative recurrence in patients with Crohn’s disease after ileocecal resection.

Category	Subcategory	Explored Risk Factors
**Clinical Factors**	**Patient-Specific**	Age, family history of inflammatory bowel disease (IBD), gender, active smoking, ethnicity, Myosteatosis (high lipid content in skeletal muscle), subcutaneous adipose tissue index, visceral adipose tissue lipid content, skeletal muscle index; micronutrient intake (isoflavones, inositol, pinitol, provitamin-A carotenoid)
	**Disease Characteristics**	Age at disease onset, duration between CD diagnosis and surgery, previous surgeries, prior use of biologics, extent of disease involvement, age at ileocecal resection, penetrating disease behavior.
**Genetic Factors**		Genetic variants including NOD2/CARD15 and CARD8 mutations.
**Surgical Factors**		Type of surgical technique, length of bowel removed, need for blood transfusions, anastomotic configuration or method, extent of mesenteric resection, perioperative complications, especially those involving intra-abdominal sepsis
**Histological Features**		Resection margin involvement, presence of granulomas, degree of transmural inflammation, evidence of myenteric or submucosal plexitis, lymphatic vessel density, S100-positive enteric glial cells
**Molecular and Metabolic Profiles**		Tissue-level transcriptomics, blood-based transcriptomics, urinary metabolomics.

**Table 3:** Risk Stratification and recommendations for managing postoperative recurrence in Crohn’s disease patients undergoing ileocecal resection.

Society	Risk Level	Criteria	Recommendation
**American Gastroenterological Association (AGA)** [5]	Low Risk	- Age > 50 years - Non-smoker - No prior ICR or surgery involving <10 cm of affected bowel - Disease duration ≥ 20 years	Endoscopic monitoring supplemented by biomarker assessments within the first year.
	High Risk	- Age < 30 years - Active smoking - ≥2 prior surgeries for penetrating or perianal disease	Initiate medical prophylaxis aimed at delaying or preventing POR, combined with endoscopic surveillance within 6–12 months.
**European Crohn’s and Colitis Organisation (ECCO)** [122]	High Risk	- Smoking - ≥1 prior intestinal surgery - Penetrating disease at the time of surgery - Perianal disease - Extensive small bowel resection (>50 cm) - Intra-Abdominal Septic Complication (IASC)	Medical prophylaxis is recommended for patients meeting any one of the high-risk criteria.
**British Society of Gastroenterology (BSG)** [94,161]	High Risk	- Active smoking - Preoperative penetrating disease behavior - Perianal involvement - History of multiple resections (≥2 prior surgeries) - Extensive bowel involvement (>50 cm)	Patients with risk factors or with preference to do so should be started on medical prophylaxis to prevent POR.

**Table 4:** Overview of studies on postoperative recurrence management strategies in Crohn’s disease.

Year	Study	Sample Size	Study Design	Intervention	Population	Key Outcomes
2015	Cruz et al. (POCER) [4]	101	RCT	Thiopurines (or adalimumab for intolerance) vs. 3-month metronidazole	High- vs. low-risk patients	Endoscopic POR rates at 18 months were similar between groups (48% vs. 56%). Treat-to-target approach with ileocolonoscopy at 6 months was superior to symptom-based management.
2015	Ferrante et al. [166]	63	RCT (terminated early)	Azathioprine vs. endoscopy-driven thiopurines at ≥i2 lesions	High-risk patients	No significant differences in endoscopic POR at 18 months; study underpowered to draw firm conclusions.
2016	Regueiro et al. (PREVENT) [9]	297	RCT	Infliximab (5 mg/kg) vs. placebo every 8 weeks for 200 weeks	High-risk patients	Clinical recurrence rates were lower in the infliximab group but not statistically significant (12.9% vs. 20.0%, *p* = 0.097). Endoscopic recurrence significantly reduced (30.6% vs. 60.0%, *p* < 0.001).
2022	Axelrad et al. [179]	1037	Retrospective	Biologic prophylaxis vs. endoscopy-driven approach	All patients	Anti-TNF within 4 weeks showed significant benefit: Any POR: aHR 0.61 (95% CI 0.40–0.93) Endoscopic POR: aHR 0.49 (95% CI 0.28–0.84) Radiographic POR: aHR 0.56 (95% CI 0.33–0.95) Anti-TNF at 4–12 weeks: Only endoscopic benefit (aHR 0.71, 95% CI 0.53–0.96) Vedolizumab and ustekinumab: No significant reduction in POR at any timepoint (but limited sample sizes)
2022	Geldof et al. [167] (PORCSE Study)	346	Retrospective	Prophylaxis (including non-biologics) vs. endoscopy-driven approach	All patients	Endoscopic POR significantly higher in the endoscopy-driven group (41.5% vs. 53.8%, OR 1.81, *p* = 0.039). as clinical POR (17.7% vs. 35.7%, OR 3.05, *p* = 0.002).
2022	Joustra et al. [168]	376	Retrospective	Prophylaxis (including non-biologics) vs. endoscopy-driven approach	High- vs. low-risk patients	Prophylaxis reduced POR only in high-risk patients. No significant difference in clinical POR at 36 months between groups.
2023	Arkenbosch et al. [180]	213	Prospective	Prophylaxis (including non-biologics) vs. endoscopy-driven approach	High- vs. low-risk patients	Endoscopic POR lower in prophylactic biologic therapy groups for both low-risk (16% vs. 45%) and high-risk (26% vs. 49%) patients.
2023	Dragoni et al. [181]	195	Retrospective	Prophylaxis (including non-biologics) vs. endoscopy-driven approach	Patients with only 1 clinical risk factor	No significant differences in endoscopic recurrence (36.1% vs. 45.5%, *p* = 0.10) or severe recurrence rates (9.8% vs. 15.7%, *p* = 0.15). Clinical POR rates also similar.
2024	Shah et al. [178]	1404	Retrospective	Biologic prophylaxis vs. endoscopy-driven approach	High-risk vs. low-risk patients	Surgical recurrence lower in high-risk patients receiving prophylaxis (10.2% vs. 16.7%, *p* = 0.02). Endoscopic POR reduced across all risk groups receiving prophylaxis.
2025	Ten Bokkel Huinink et al. [182]	807	Retrospective	Prophylaxis (including non-biologics) vs. endoscopy-driven approach	All patients	Surgical recurrence: 11% (prophylaxis) vs. 17% (no prophylaxis), *p* = 0.01 Severe endoscopic recurrence (mRS ≥ i3): 20% vs. 34%, *p* < 0.01 Endoscopic/radiologic recurrence: 41% vs. 61%, *p* < 0.01

**Table 5:** Pharmacologic strategies investigated to prevent postoperative recurrence in patients with Crohn’s disease undergoing ileocecal resection. Modified from Ferrante et al. [8].

Year	Authors	Study Type	Sample Size (*n*)	Intervention	Findings
Antibiotics					
1995	Rutgeerts et al. [227]	RCT	60	Metronidazole (20 mg/kg/day) vs. placebo	Reduced endoscopic POR (52% vs. 75%, *p* = 0.09), severe endoscopic POR (13% vs. 43%, *p* = 0.02), and histological POR (17% vs. 54%, *p* = 0.008) at 3 months.
2005	Rutgeerts et al. [248]	RCT	80	Ornidazole (1 g/day) vs. placebo	Decreased clinical (8% vs. 38%, *p* = 0.0046) and endoscopic POR (54% vs. 79%, *p* = 0.037) at 1 year.
2013	Herfarth et al. [249]	RCT	33	Ciprofloxacin (500 mg twice daily) vs. placebo	Similar endoscopic POR rates (65% vs. 69%, *p* < 0.805) at 6 months.
Mesalamine					
2015	Singh et al. [231]	Network meta-analysis	811	Mesalamine/sulfasalazine vs. placebo	Reduced clinical POR (RR 0.60, 95% CI 0.37–0.88).
2015	Singh et al. [231]	Network meta-analysis	766	Mesalamine/sulfasalazine vs. placebo	No significant reduction in endoscopic POR (RR 0.67, 95% CI 0.39–1.08).
2019	Gjuladin-Hellon et al. [250]	Meta-analysis	730	Mesalamine vs. placebo	Reduced clinical POR (36% vs. 43%, RR 0.83, 95% CI 0.72–0.96) over 12–72 months.
2019	Gjuladin-Hellon et al. [250]	Meta-analysis	537	Mesalamine vs. placebo	Similar endoscopic POR (70% vs. 73%, RR 0.83, 95% CI 0.56–1.23) over 12–72 months.
Corticosteroids					
1999	Ewe et al. [251]	RCT	62	Budesonide (1 mg 3×/day) vs. placebo	No significant difference in clinical and/or endoscopic POR (57% vs. 70%, *p* = ns) at 1 year.
1999	Hellers et al. [252]	RCT	129	Budesonide (6 mg/day) vs. placebo	No significant difference in endoscopic POR at 3 months (31% vs. 52%, *p* = ns) or 12 months (52% vs. 58%, *p* = ns).
Immunomodulators					
2009	Peyrin-Biroulet et al. [253]	Meta-analysis	433	Thiopurines vs. placebo/mesalamine/metronidazole	Reduced clinical POR (mean difference 8%, 95% CI: 1–15%, *p* = 0.021).
2009	Peyrin-Biroulet et al. [253]	Meta-analysis	293	Thiopurines vs. placebo/mesalamine/metronidazole	Reduced endoscopic POR (mean difference 15%, 95% CI 1.8–29%, *p* = 0.026).
2019	Gjuladin-Hellon et al. [254]	Meta-analysis	408	Thiopurines vs. placebo	Reduced clinical POR (51% vs. 64%, RR 0.79, 95% CI 0.67–0.92) over 12–36 months.
2019	Gjuladin-Hellon et al. [254]	Meta-analysis	321	Thiopurines vs. placebo	No significant reduction in endoscopic POR (67% vs. 75%, RR 0.85, 95% CI 0.64–1.13) over 12–36 months.
Anti-TNFα					
2009	Regueiro et al. [234]	RCT	24	Infliximab vs. placebo	Reduced endoscopic POR (9.1% vs. 84.6%, *p* = 0.0006) and histologic POR (27.3% vs. 84.6%, *p* = 0.01) at 1 year. Clinical remission was not significantly different (80% vs. 53.8%, *p* = 0.38).
2012	Yoshida et al. [255]	RCT	31	Infliximab and immunomodulator/corticosteroid vs. immunomodulator/corticosteroid alone	12-month and 36-month clinical remission, defined by CDAI < 150, were significantly higher in patients receiving infliximab compared with placebo (100% and 93% vs. 69% and 56%, respectively, *p* < 0.03)
2013	Savarino et al. [165]	RCT	51	Adalimumab vs. thiopurines vs. mesalamine	Adalimumab showed significantly lower endoscopic POR (6.3%) compared to azathioprine (64.7%, OR 0.036, 95% CI 0.004–0.347) and mesalamine (83.3%, OR 0.013, 95% CI 0.001–0.143) at 2 years. Clinical recurrence was also lower with adalimumab (12.5%) vs. azathioprine (64.7%, OR 0.078, 95% CI 0.013–0.464) and mesalamine (50%, OR 0.143, 95% CI 0.025–0.819).
2016	Regueiro et al. [9]	RCT	297	Infliximab vs. placebo	No significant difference in clinical POR (12.9% vs. 20%, *p* = 0.097), but reduced endoscopic POR (30.6% vs. 60%, *p* < 0.002) at 76 weeks.
2022	Beelen et al. [230]	Meta-analysis	645	Anti-TNFα vs. thiopurines	There was no statistically significant difference in clinical POR between groups (12.9% vs. 20%, *p* = 0.097), but endoscopic POR was significantly lower (30.6% vs. 60%, *p* < 0.002) at 76 weeks. Anti-TNFα therapy demonstrated greater efficacy than thiopurine prophylaxis in reducing endoscopic POR (RR 0.52; 95% CI 0.33–0.80), clinical POR (RR 0.50; 95% CI 0.26–0.96), and severe endoscopic POR (RR 0.41; 95% CI 0.21–0.79).
Ustekinumab					
2021	Buisson et al. [244]	Retrospective cohort	63	Ustekinumab vs. azathioprine	Lower endoscopic POR (28% vs. 54.5%, *p* = 0.029) at 6 months.
2022	Manosa et al. [256]	Retrospective cohort	40	Vedolizumab vs. Ustekinumab	Clinical POR in 32% at 12 months and endoscopic POR in 42% within 18 months.
2022	Yanai et al. [242]	Retrospective cohort	297	Ustekinumab and vedolizumab vs. Anti-TNFα	Overall endoscopic POR was 41.8% at 1 year, with no significant difference between ustekinumab and Anti-TNFα (OR 1.86, 95% CI 0.79–4.38).
Vedolizumab					
2018	Yamada et al. [257]	Retrospective cohort	80	Vedolizumab vs. Anti-TNFα	Lower endoscopic remission rates at 6–12 months compared to Anti-TNFα (25% vs. 66%, *p* = 0.01).
2022	Manosa et al. [256]	Retrospective cohort	25	Vedolizumab vs. Ustekinumab	Clinical POR in 30% at 12 months and endoscopic POR in 40% within 18 months.
2022	Yanai et al. [242]	Retrospective cohort	297	Ustekinumab and vedolizumab vs. Anti-TNFα	Overall endoscopic POR was 41.8% at 1 year, with no significant difference between vedolizumab and Anti-TNFα (OR 0.55, 95% CI 0.25–1.19).
2025	D’Haens et al. [243]	RCT	84	Vedolizumab vs. placebo	Significant reduction in severe endoscopic POR (23.3% vs. 62.2%, *p* < 0.0004) at 76 weeks.

**Table 6:** Comparative outcomes of therapies in managing postoperative recurrence in patients with Crohn’s disease undergoing ileocecal resection. Modified from Ferrante et al. [8].

Study	Date	Study Design	Sample Size	Intervention	Key Findings
Mesalamine					
Reinisch et al. [261]	2010	RCT	78	Mesalamine vs. azathioprine	No significant difference in treatment failure (11% vs. 22%, *p* = 0.19) at year 1. Clinical POR more common with mesalamine; endoscopic improvement less frequent (34.4% vs. 63.3%, *p* = 0.023).
Orlando et al. [229]	2020	RCT	46	Mesalamine vs. azathioprine	No significant difference in treatment failure (21% vs. 14%, *p* = 0.702) at year 1. Clinical POR more frequent with mesalamine; endoscopic improvement lower (8.3% vs. 36.4%, *p* = 0.035).
Thiopurines					
Reinisch et al. [261]	2010	RCT	78	Mesalamine vs. azathioprine	Azathioprine associated with less clinical POR and more frequent endoscopic improvement (34.4% vs. 63.3%, *p* = 0.023). No significant difference in treatment failure (11% vs. 22%, *p* = 0.19).
Orlando et al. [229]	2020	RCT	46	Mesalamine vs. azathioprine	Azathioprine resulted in less clinical POR and greater endoscopic improvement (8.3% vs. 36.4%, *p* = 0.035). No significant difference in treatment failure (21% vs. 14%, *p* = 0.702).
Anti-TNFα agents					
Cañete et al. [262]	2020	Retrospective Cohort	179	Infliximab or adalimumab	Endoscopic improvement in 61%, including 42% achieving remission. Better outcomes with infliximab vs. adalimumab and concomitant thiopurine use; no impact of preoperative Anti-TNFα exposure.
Carla-Moreau et al. [263]	2015	Meta-analysis (2 studies)	50	Infliximab vs. control arms (azathioprine/mesalamine)	Infliximab significantly more effective for treating endoscopic POR (OR 16.64; 95% CI 2.51–110.27).
Ustekinumab					
Tursi et al. [264]	2021	Retrospective Case-Series	15	Observational	Clinical remission in 12/15 patients at a median of 6 months; all 11 patients undergoing colonoscopy achieved endoscopic remission.
Macaluso et al. [259]	2023	Retrospective Case-Series	44	Observational (mean follow-up: 18 months)	Clinical remission in 32/44 patients; endoscopic improvement in 22/44; endoscopic remission in 12/44.
Vedolizumab					
Macaluso et al. [265]	2022	Retrospective Cohort	58	Observational (mean follow-up: 25 months)	Endoscopic improvement in 48% (mean time: 16 months); clinical failure in 19% at 1 year; 12% required a new resection.

## Data Availability

No new data were created or analyzed in this study. Data sharing is not applicable to this article.

## Abbreviations

The following abbreviations are used in this manuscript:

5-ASA	5-aminosalicylic acid
AGA	American Gastroenterological Association
aHR	adjusted hazard ratio
aOR	adjusted odds ratio
AUC	area under the curve
AUROC	area under the receiver operating characteristic curve
BAM	bile acid malabsorption
BSG	British Society of Gastroenterology
BWT	bowel wall thickness
CD	Crohn’s disease
CDAI	Crohn’s Disease Activity Index
CI	confidence interval
CRP	C-reactive protein
CTE	computed tomography enterography
CTLs	cytotoxic T lymphocytes
CXCL9	C-X-C motif chemokine ligand 9
ECCO	European Crohn’s and Colitis Organisation
EHI	Endoscopic Healing Index
FC	fecal calprotectin
HBI	Harvey-Bradshaw Index
HDL	high-density lipoprotein
HR	hazard ratio
IASC	intra-abdominal septic complication
IBD	inflammatory bowel disease
IBS	irritable bowel syndrome
ICR	ileocecal resection
IFN-γ	interferon-gamma
IGF-1	insulin-like growth factor 1
IL	interleukin
IUS	intestinal ultrasound
LDL	low-density lipoprotein
MaRIA	Magnetic Resonance Index of Activity
MRE	magnetic resonance enterography
MRI	magnetic resonance imaging
NKG2D	natural killer group 2D
NOD2	nucleotide-binding oligomerization domain-containing protein 2
NPV	negative predictive value
OR	odds ratio
POR	postoperative recurrence
PPV	positive predictive value
RCT	randomized controlled trial
RR	relative risk
SES-CD	Simple Endoscopic Score for Crohn’s Disease
SUCRA	surface under the cumulative ranking curve
TCR	T-cell receptor
TGF-β1	transforming growth factor beta 1
Th1	T helper 1
Th17	T helper 17
TNF	tumor necrosis factor
